# GOLPH3/CKAP4 promotes metastasis and tumorigenicity by enhancing the secretion of exosomal WNT3A in non-small-cell lung cancer

**DOI:** 10.1038/s41419-021-04265-8

**Published:** 2021-10-21

**Authors:** Jun-Wei Song, Jing Zhu, Xing-Xuan Wu, Ting Tu, Jing-Qiang Huang, Guan-Zi Chen, Li-Yin Liang, Chun-Hui Zhou, XingZhi Xu, Li-Yun Gong

**Affiliations:** 1 GuangDong Key Laboratory for Genome Stability and Human Disease Prevention, Department of Biochemistry and Molecular Biology, Health Science Center, Shenzhen University, 518060 Shenzhen, Guangdong P. R. China; 2 Department of Pathogen Biology, Guangdong Provincial Key Laboratory of Regional Immunity and Diseases, Health Science Center, Shenzhen University, 518060 Shenzhen, Guangdong P. R. China; 3 Department of Cell Biology and Medical Genetics, School of Basic Medical Sciences, Health Science Center, Shenzhen University, 518055 Shenzhen, Guangdong China; 4 Guangzhou Health Science College, 510520 Guangzhou, Guangdong P. R. China; 5 Shenzhen University-Friedrich Schiller Universität Jena Joint PhD Program in Biomedical Sciences, Health Science Center, Shenzhen University, 518055 Shenzhen, Guangdong China; 6 Carson International Cancer Center, Health Science Center, Shenzhen University, 518055 Shenzhen, Guangdong China

**Keywords:** Tumour biomarkers, Cell invasion

## Abstract

Cancer metastasis is the main cause of mortality associated with non-small-cell lung cancer (NSCLC), accounting for up to 70% of deaths among patients. The mechanisms underlying distal metastasis remain largely unknown. Golgi phosphoprotein 3 (GOLPH3) correlates negatively with overall survival in multiple tumors. In this study, we evaluated the function of GOLPH3 in NSCLC distal metastasis. GOLPH3 was expressed at high levels in samples from patients with NSCLC and was positively associated with clinicopathologic characteristics including clinical stage (*P* < 0.001), T (*P* = 0.001), N (*P* = 0.007), and M (*P* = 0.001) classification. Functionally, Transwell and wound-healing assays suggested that *GOLPH3* overexpression enhances NSCLC cell migration and invasion abilities. Tumor-sphere formation and flow cytometry assays demonstrated that GOLPH3 overexpression enhances a stem cell-like phenotype of NSCLC cells. Metastasis models established by tail vein and intracardiac injection confirmed the pro-metastatic function of GOLPH3 in vivo. A subcutaneous tumor formation model confirmed that *GOLPH3* overexpression increased the tumorigenicity of NSCLC cells. Mechanistically, gene set enrichment analysis revealed a positive association of *GOLPH3* mRNA expression with WNT-activated gene signatures. Luciferase-reporter and nuclear extract assays showed that *GOLPH3* overexpression enhances metastasis and tumorigenicity through activation of the WNT/β-catenin pathway. Immunoprecipitation-mass spectrometry and gene ontology analysis demonstrated that GOLPH3 interacts with cytoskeleton-associated protein 4 (CKAP4) in exosome-mediated distal metastasis. We found that GOLPH3 decreased the amount of plasma membrane-localized CKAP4 and increased the amount of exosome-localized CKAP4 to promote the formation of CKAP4-containing exosomes. Furthermore, we demonstrated that CKAP4 binds exosomal WNT3A to enhance its secretion. Therefore, the GOLPH3/CKAP4 axis plays a crucial role in promoting exosomal-WNT3A secretion to enhance and maintain the stem-like phenotype and metastasis in NSCLC, thus indicating the therapeutic potential of GOLPH3 in patients with NSCLC metastasis.

## Introduction

Lung cancer has a high rate of mortality and remains the second most commonly diagnosed cancer worldwide [1]. Based on clinical histology, ~85% of lung cancer cases are diagnosed as non-small cell lung cancer (NSCLC) with an overall 5-year survival rate of only 15% [2]. It has been reported that metastasis is responsible for >70% of NSCLC deaths, and the majority of patients with advanced-stage NSCLC survive for only 18 months after diagnosis [3]. Thus, determining the mechanism of distal metastasis is important to improve the poor prognosis of patients with NSCLC and to identify biomarkers or drug targets.

Tumor metastasis is a complex multistep process that includes local invasion by tumor cells, evasion of immune surveillance, intravasation into the circulation, and extravasation at secondary organs to form macroscopic metastatic lesions [4]. Exosomes are nanosized membrane vesicles (diameter 30–150 nm) that contain cytosolic proteins, transport proteins, lipids, and nucleic acid, such as miRNAs and lncRNA [5]. Numerous studies have suggested that exosomes are critical for all steps of tumor metastasis. Wang et al. found that exosomal miR-105 targets the mRNA encoding the tight junction protein ZO-1 to induce destruction of tight junctions, which act as natural barriers to the movement of cells that lead to metastasis of breast cancer [6]. Hoshino et al. revealed that the exosomal integrins α6β4 and α6β1 enhance the tropism of lung metastases, while exosomal integrin αvβ5 was more closely associated with the tropism of liver metastases. Exosomal miR-9 targets and effectively activates the JAK/STAT pathway to increase endothelial cell migration and tumor angiogenesis [7]. The exosomal lncRNA *FMR1-AS1* interacts with TLR7, leading to enhanced expression of c-myc, thus maintaining cancer stem-like cell dynamics and inducing esophageal squamous-cell carcinoma cell invasion ability [8]. Despite extensive research on exosomal nucleic acids, the role of secretory proteins in exosomes is poorly understood.

The WNT/β-catenin signaling pathway plays a pivotal role in carcinogenesis and the stem cell-like phenotype of NSCLC cells [9]. WNT ligands bind to low-density lipoprotein receptor-related protein (LRP)5/6 and frizzled co-receptors to drive reprogramming of gene expression that supports stem cell maintenance, metastasis, and differentiation [10]. Mao et al. found that the exosomes derived from p53^−/−^ mouse bone marrow mesenchymal stem cells transferred ubiquitin protein ligase E3 component N-recognin 2 (UBR2) to regulate the WNT/β-catenin signaling that induces gastric cancer growth and metastasis [11]. WNTs, which are lipids modified by the acyltransferase porcupine, are cysteine-rich glycoproteins capable of functioning in both paracrine and autocrine manners [12, 13]. Recent studies showed that WNT proteins are carried on the surface of secreted exosomes that migrate to target cells to activate the WNT signaling pathway [14]. Exosomal WNT11 binds frizzled (FZD)-disheveled segment polarity protein 1 (DVl) receptors to activate the WNT-prolylcarboxypeptidase (PCP) pathway, resulting in enhanced breast cancer cell protrusive activity, motility, and metastatic potential [15].

Golgi phosphoprotein 3 (GOLPH3) is a peripheral membrane protein that is highly localized to the trans-Golgi, where it is involved in vesicle budding and anterograde and retrograde Golgi trafficking [16, 17]. Studies suggest that GOLPH3 is highly expressed in numerous cancers and related to proliferation, tumorigenicity, and drug resistance [17, 18]. Moreover, some studies showed exosomes originate from the trans-Golgi network or internalized membrane microdomains, with biogenesis initiated by early endosome (EE) formation [19, 20]. Thus, we hypothesized that GOLPH3 participates in the regulation of exosomal protein secretion, leading to cancer progression. Therefore, we investigated the involvement of GOLPH3 in NSCLC progression. We showed that GOLPH3 interacts with cytoskeleton-associated protein 4 (CKAP4) to enhance the secretion of exosomal WNT3A, leading to a stem cell-like phenotype and metastasis in NSCLC.

## Results

### Elevated *GOLPH3* expression is associated with the clinicopathologic characteristics of NSCLC

**Fig. 1:**
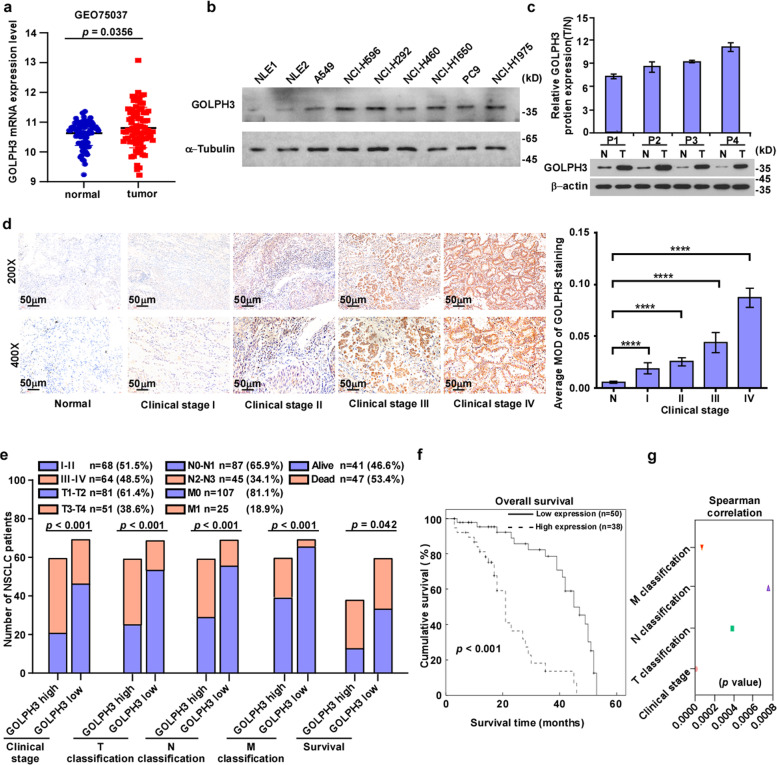
Elevated expression of GOLPH3 is associated with the clinicopathologic characteristics of NSCLC. **a** Analysis of *GOLPH3* expression in a publicly available NSCLC dataset (NCBI/GEO/GSE75037). **b** Western blot analysis of GOLPH3 protein levels in the NLEC and NSCLC cell lines. **c** Quantification (top) and western blot analysis (bottom) of GOLPH3 protein expression in primary NSCLC tissues (T) and paired adjacent noncancerous lung tissues (N). Data represent the mean ± SD of three independent experiments. **d** IHC staining (left) and statistical analyses (right) of the average MOD of GOLPH3 expression in NSCLC specimens (magnification: upper ×200; lower ×400). Data represent the mean ± SD of three independent experiments; ***P < 0.001. **e** GOLPH3 overexpression correlates significantly with NSCLC clinical features (clinical stage, TNM classification, or survival). **f** Kaplan–Meier analysis of overall survival by GOLPH3 expression in NSCLC. **g** Spearman correlation analyses of GOLPH3 expression and clinical stage or TNM classification.

Analysis of a publicly available NSCLC dataset (NCBI/GEO/GSE75037) revealed that *GOLPH3* mRNA levels were significantly higher in NSCLC cancer tissues compared with those in normal human lung tissues (Fig. 1a). Furthermore, western blot analysis showed a higher level of GOLPH3 in NSCLC cell lines than those in two normal lung epithelial cell lines (Fig. 1b). Moreover, GOLPH3 protein expression levels were significantly higher in the NSCLC tissues compared with those in the tumor-adjacent lung tissues (Fig. 1c). To explore the clinical role of GOLPH3 expression in NSCLC progression, we used IHC staining to examine GOLPH3 expression in 132 formaldehyde-fixed, paraffin-embedded NSCLC samples, which included 68 cases with clinical stage I–II lesions (51.5%), and 64 cases with stage III–IV lesions (48.5%) (Fig. 1d, e). The average mean optical density (MOD) of GOLPH3 staining indicated that GOLPH3 levels increased with the clinical stage progression in NSCLC tissues (Fig. 1d). Kaplan–Meier analysis revealed that GOLPH3 expression was negatively associated with overall survival (*P* < 0.001; Fig. 1f). We then analyzed the differences between the survival curves of patients with high and low GOLPH3 expression in subgroups of clinical stage and tumor-node metastasis (TNM) classification (Supplementary Fig. 1a–h). This analysis indicated that GOLPH3 protein expression level was positively associated with clinical stage (*P* < 0.001), and T (*P* < 0.001), N (*P* < 0.001), and M (*P* < 0.001) classification in patients with NSCLC (Fig. 1g). Thus, these data suggest that high GOLPH3 expression enhances NSCLC metastasis and correlates significantly and negatively with overall survival time.

### Elevated *GOLPH3* expression enhances the migration and invasion, as well as the stem cell-like phenotype, of NSCLC in vitro

**Fig. 2:**
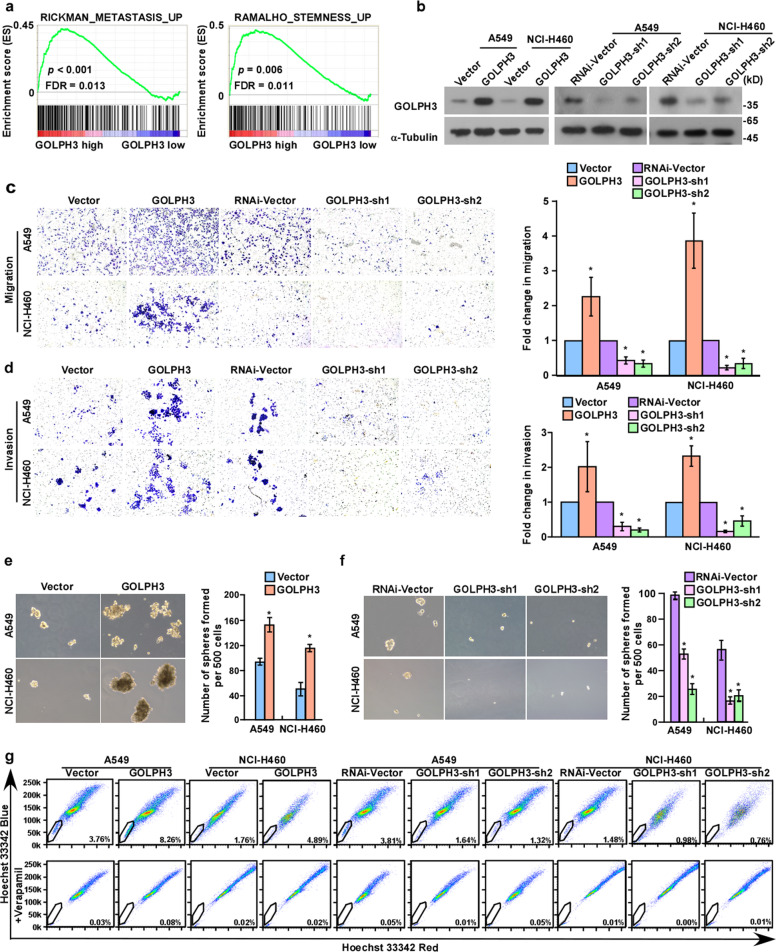
Elevated GOLPH3 expression enhances the migration/invasion abilities and stem cell-like phenotype of NSCLC cells in vitro. **a** GSEA indicates that *GOLPH3* expression is positively associated with metastasis and stemness signatures (RICKMAN_METASTASTS_UP; RAMALH_ STEMNESS_UP). **b** Western blot analysis of GOLPH3 expression in the indicated cell lines stably expressing *GOLPH3*. **c** Migration assay of the indicated cell lines stably expressing *GOLPH3* (left). The ratio of cells migrated across the Transwell membrane (right). **d** Invasion assay of cell lines stably expressing *GOLPH3* (left). The ratio of cells invading the Matrigel in Transwell chambers (right). **e** Representative micrographs of tumor-sphere formation by *GOLPH3*-overexpressing or vector-transduced cells (left). Number of tumor spheres formed by the indicated cells (right). **f** Representative micrographs of tumor-sphere formation by *GOLPH3*-silenced or control cells (left). Number of tumor spheres formed by the indicated cells (right). **g** Flow cytometric analysis of the proportion of SP^+^ NSCLC cells. Data represent the mean ± SD of three independent experiments; *P < 0.05.

Gene set enrichment analysis (GSEA) of a publicly available NSCLC dataset (NCBI/GEO/GSE75037) showed that *GOLPH3* expression was positively associated with metastasis and cancer stem cell-like phenotype (Fig. 2a). To explore the biological functions of GOLPH3, we successfully established stable A549 and NCI-H460 NSCLC cell lines with overexpression and silenced expression of *GOLPH3* (Fig. 2b). Furthermore, Transwell assays of *GOLPH3*-overexpressing and *GOLPH3*-silenced cells suggested that the migratory and invasive abilities of NSCLC cells were strongly increased in the GOLPH3-overexpressing cell lines (Fig. 2c, d). Wound-healing assays revealed that the speed of wound closure was increased for *GOLPH3*-overexpressing NSCLC cells, but was reduced for GOLPH3-silenced cells (Supplementary Fig. 2a). Real-time PCR analysis of stemness-related markers revealed that mRNA levels of ABCG2, C-MYC, CD133, NANOG, and KLF4 were significantly upregulated in GOLPH3-overexpressing cells, while the levels were downregulated in *GOLPH3*-silenced NSCLC cells. These observations indicated that high GOLPH3 expression enhances the mRNA levels of stemness-related markers in NSCLC cells (Supplementary Fig. 2b). Moreover, tumor-sphere formation assays showed that *GOLPH3*-overexpressing cells formed a greater number of spheres containing higher cell numbers compared with those formed by the control cells, whereas *GOLPH3*-silenced cells showed lower tumor-sphere formation ability compared with the control cells (Fig. 2e, f). Moreover, we found that *GOLPH3* overexpression enhanced the proportion of A549 SP^+^ cells from 3.76 to 8.26%, and the proportion of NCI-H460 SP^+^ cells from 1.76 to 4.89%. Conversely, low expression of *GOLPH3* decreased the proportion of A549 SP^+^ cells from 3.81 to 1.32%, and the proportion of NCI-H460 SP^+^ cells from 1.48 to 0.76% (Fig. 2g). Therefore, these data indicated that *GOLPH3* overexpression enhances metastasis and the cancer stem cell-like subpopulation to promote a cancer stem cell-like phenotype in NSCLC cells in vitro.

### *GOLPH3* overexpression promotes distal metastasis in vivo

**Fig. 3:**
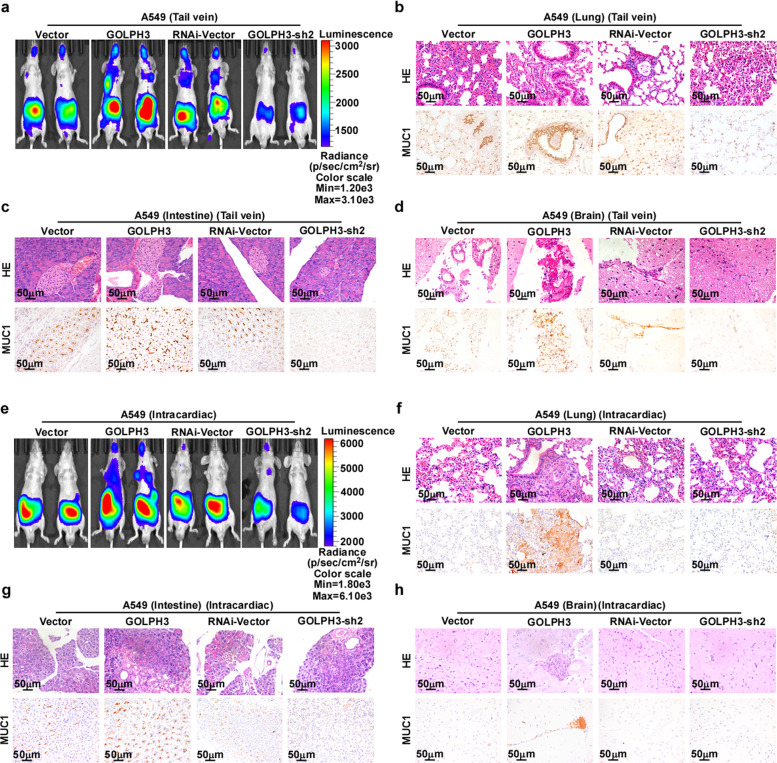
GOLPH3 overexpression promotes distal metastasis of NSCLC xenografts in vivo. **a** For the intravenous (tail vein) injection metastasis model, bioluminescent images of subcutaneous tumors generated from the indicated cells and distant metastasis signals are shown. **b**–**d** Histological confirmation of tumor cells by H&E staining. Representative images of an IHC assay of the lung adenocarcinoma marker (MUC1) in metastatic lesions in the lung (**b**), intestine (**c**), and brain (**d**). **e** For the intracardiac injection transfer model, bioluminescent images of subcutaneous tumors of A549-luc-vector, A549-luc-GOLPH3, A549-luc-RNAi-vector, and A549-luc-GOLPH3-sh2; distant metastasis signals are also shown. **f**–**h** Histological confirmation of tumor cells by H&E staining. Representative images of an IHC assay of MUC1 in metastatic lesions in the lung (**f**), intestine (**g**), and brain (**h**).

To clarify the role of GOLPH3 in NSCLC metastasis in vivo, we established a mouse model by intravenous injection of A549-luc-vector, A549-luc-GOLPH3, A549-luc-RNAi-vector, or A549-luc-GOLPH3-shRNA#2 cells (1 × 10^6^) into the tail vein. Only injection of the A549-luc-GOLPH3 cells resulted in a visible metastasis signal throughout almost the whole body, whereas metastatic lesions detected following injection of A549-luc- GOLPH3-shRNA#2 cells were limited to the intestine (Fig. 3a). IHC analysis suggested that the metastatic lesions induced in multiple organs following injection of A549-related cells were positive for mucin 1 (MUC1) (Fig. 3b–d), thus confirming that these lesions originated from the inoculated NSCLC cells. This metastasis model showed that *GOLPH3* overexpression induced NSCLC cell metastasis at distal organs. To confirm the metastatic function of GOLPH3 in vivo, we constructed an intracardiac injection transfer model in mice. Bioluminescence imaging showed that mice bearing A549-luc-GOLPH3 tumors following injection of 1 × 10^6^ cells exhibited spontaneous metastatic colonization of the lung, intestines, and brain. In contrast, spontaneous metastatic colonization of the intestine alone was detected in the vector control group (Fig. 3e). We also found that A549-luc-RNAi-vector and A549-luc-GOLPH3-shRNA#2 cells caused only limited intestinal metastases, but the GOLPH3 low-expression group had weaker metastasis signals compared with the control group (Fig. 3e). IHC analysis revealed that the metastatic lesions in the lung and multiple organs were MUC1-positive, indicating that these lesions developed from intracardiac injection of A549-luc-GOLPH3 cells (Fig. 3f–h). Taken together, these results indicated that elevated *GOLPH3* expression confers metastatic capability on NSCLC cells in vivo.

### *GOLPH3* overexpression enhances the tumorigenicity of NSCLC cells in vivo

**Fig. 4:**
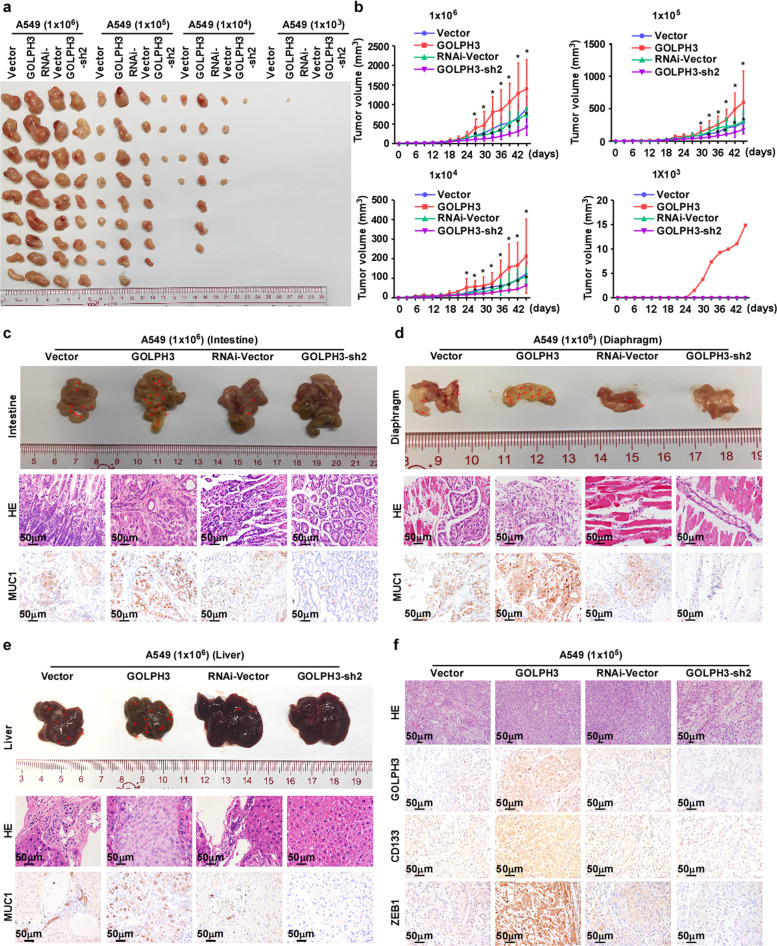
GOLPH3 overexpression enhances the tumorigenicity and metastasis of NSCLC cells in vivo. **a** Tumors formed by different numbers of A549 cells (1 × 10^6^, 1 × 10^5^, 1 × 10^4^, or 1 × 10^3^) transduced with pMSCV-GOLPH3, pMSCV-vector, pSuper-GOLPH3-shRNA#1, or pSuper-vector plasmid. **b** Tumor growth curves following implantation of 1 × 10^6^, 1 × 10^5^, 1 × 10^4^, or 1 × 10^3^ cells. **c**–**e** Histological confirmation of tumor cells by H&E staining. Representative images of an IHC assay of MUC1 in metastatic lesions in the intestine (**c**), diaphragm (**d**), and liver (**e**). **f** Histological confirmation of tumor cells by H&E staining. Representative images of an IHC assay of GOLPH3, CD133, and ZEB1 in subcutaneous tumors formed by the indicated cells.

To explore the role of GOLPH3 in promoting a tumor stemness phenotype in NSCLC cells in vivo, we injected A549 cells (1 × 10^6^, 1 × 10^5^, 1 × 10^4^ or 1 × 10^3^) mixed with Matrigel subcutaneously into the inguinal folds of BALB/c nude mice. In all cases, we found that *GOLPH3*-overexpressing A549 cells formed larger tumors than those formed by the vector control cells (Fig. 4a, b and Supplementary Fig. 2c). Conversely, tumors formed by the *GOLPH3*-silenced cell lines were smaller than those formed by the vector control cells (Fig. 4a, b). Our results suggested that elevated *GOLPH3* expression significantly increased the tumorigenicity of NSCLC cells (Supplementary Fig. 2c). Importantly, tumors were formed by *GOLPH3*-overexpressing A549 cells only when 1 × 10^3^ cells were implanted (Fig. 4a). Interestingly, we found that mice bearing A549-GOLPH3 tumors derived from 1 × 10^6^ cells exhibited more spontaneous metastatic colonization in multiple distal organs compared with that of the control group. IHC analysis revealed that the experimental metastatic lesions in the liver, diaphragm, and intestine were MUC1-positive, suggesting that these lesions developed from subcutaneously inoculated A549-GOLPH3 cells (Fig. 4c–e). Furthermore, IHC analysis showed high expression of GOLPH3, CD133, and ZEB1 in the tumor tissues formed by A549-GOLPH3 cells (Fig. 4f). These data suggested that high *GOLPH3* expression promotes the stemness phenotype of NSCLC cells and enhances NSCLC metastasis in vivo.

### Exosomes derived from *GOLPH3*-overexpressing cells regulate NSCLC metastasis and the stem cell-like phenotype through activation of the WNT/β-catenin signaling pathway

**Fig. 5:**
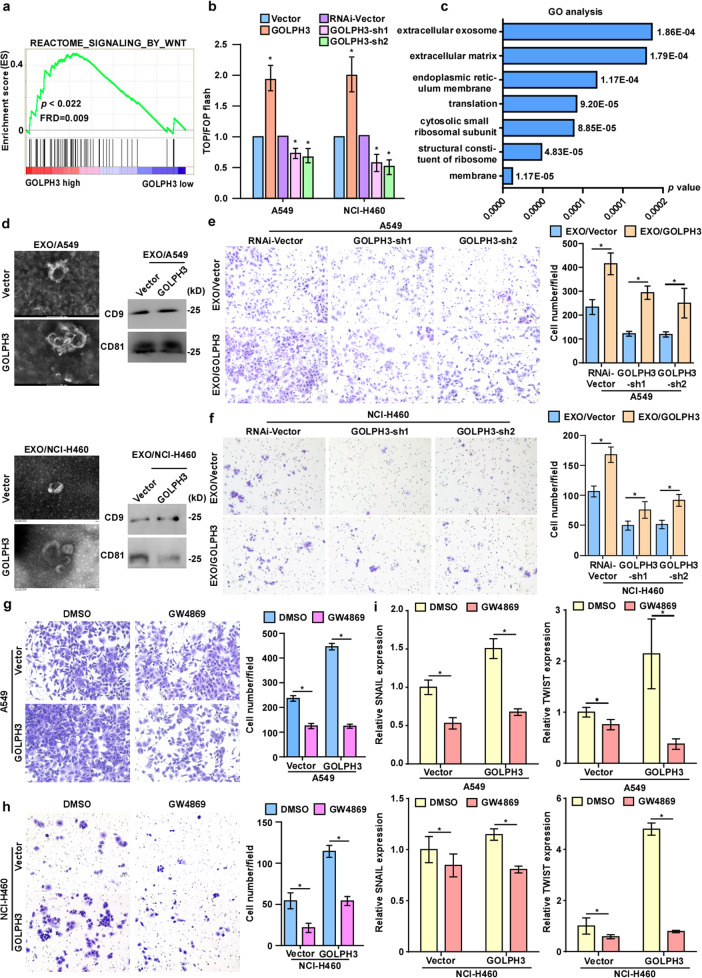
Exosomes derived from GOLPH3-overexpressing cells regulate NSCLC metastasis and a stem cell-like phenotype via activation of the WNT/β-catenin signaling pathway. **a** GSEA of publicly available NSCLC profiles (REACTOME_SIGNALING_BY_WNT) suggests that *GOLPH3* mRNA expression is associated positively with the activation of WNT signaling. **b** Luciferase-reporter assays of TOP/FOP transcriptional activity in the indicated NSCLC cells. **c** GO analysis of differentially expressed genes identified by IP-MASS analysis of SFB-vector and SFB-GOLPH3 cells. **d** Electron micrograph of exosomes isolated from *GOLPH3*-overexpressing and vector control cells (left; scale bar, 200 nm). Western blot analysis of CD9 and CD81 levels in exosomes of *GOLPH3*-overexpressing and vector control cells (right). **e**, **f** Migration assay of the *GOLPH3*-silenced cell line treated with exosomes from A549-GOLPH3 cells (**e**) or NCI-H460-GOLPH3 cells (**f**) (left). The number of cells that migrated to the lower Transwell chamber, *P < 0.05, right. **g**, **h** Migration assay of A549-GOLPH3 cells (**g**) and NCI**-**H460-GOLPH3 cells (**h**) treated with GW4869 (left). Number of cells that migrated to the lower Transwell chamber; *P < 0.05, right. **i** Real-time PCR analysis of the mRNA expression of metastasis-related markers in GOLPH3-overexpressing and vector cells treated with GW4869.

To explore the mechanism by which GOLPH3 regulates the cancer stem cell-like phenotype and promotes metastasis in NSCLC, we used GSEA software programs to examine the relationship between the expression of *GOLPH3* and the genes regulated by various signaling signatures. *GOLPH*3 mRNA expression correlated positively with WNT-activated gene signatures (Fig. 5a), indicating that high GOLPH3 expression enhances activation of the WNT/β-catenin signaling pathway. Furthermore, luciferase-reporter assays suggested that *GOLPH3* overexpression markedly increased the transcriptional activation of *TCF/LEF* genes in the A549 and H460 cells, but was attenuated by *GOLPH3* knockdown (Fig. 5b). Nuclear extraction and immunofluorescence assays suggested that *GOLPH3* overexpression substantially increased the accumulation of β-catenin in the nucleus, but was reduced by *GOLPH3* knockdown (Supplementary Fig. 3a–c). Furthermore, real-time PCR analysis showed that GOLPH3 overexpression enhanced the mRNA levels of the downstream targets of WNT/β-catenin signaling, including *C-MYC*, *TWIST*, *CCND1*, *SNAIL*, and *CD44*, whereas expression of these targets was decreased by GOLPH3 knockdown (Supplementary Fig. 3d, e). Overall, these data suggested that GOLPH3 overexpression promotes activation of the WNT/β-catenin signaling pathway.

To explore the mechanism by which GOLPH3 regulates WNT/β-catenin signaling, we employed an immunoprecipitation and mass spectrometry (IP-MASS) approach to identify GOLPH3-interacting proteins in 293T cells. Gene ontology (GO) analysis of the differentially expressed genes identified in the IP-MASS analysis of cells transfected with SFB-vector and SFB-GOLPH3 showed that GOLPH3 predominantly binds proteins associated with exosome secretion (Fig. 5c). We then extracted exosomes from *GOLPH3*-overexpressing and vector control cells and confirmed the structural features of exosomes by phase-contrast electron microscopy and nanoparticle tracking analysis (NTA) (Supplementary Fig. 4a–d). Expression of the exosome markers CD9 and CD81 was verified by western blot analysis (Fig. 5d). Transwell migration assays also showed that treatment with exosomes secreted from *GOLPH3*-overexpressing cells rescued the migration ability of GOLPH3-sh1 and sh2 cells compared with the effects of treatment with exosomes secreted from vector-transduced cells (Fig. 5e, f). We also used Transwell migration assays to explore the migratory ability of GOLPH3-overexpressing and vector-transduced cells treated with the exosome generation inhibitor GW4869. Treatment with GW4869 inhibited the metastasis of NSCLC cells induced by high expression of *GOLPH3* (Fig. 5g, h). Furthermore, real-time PCR analysis showed that GW4869 treatment inhibited the metastasis and stem cell-like phenotype of NSCLC cells induced by high *GOLPH3* expression (Fig. 5i and Supplementary Fig. 5a). Thus, our findings showed that exosomes derived from GOLPH3-overexpressing cells enhance the metastasis and stem cell-like phenotype of NSCLC cells.

### WNT3A in exosomes derived from GOLPH3-overexpressing cells enhances NSCLC cell metastasis and the stem cell-like phenotype

**Fig. 6:**
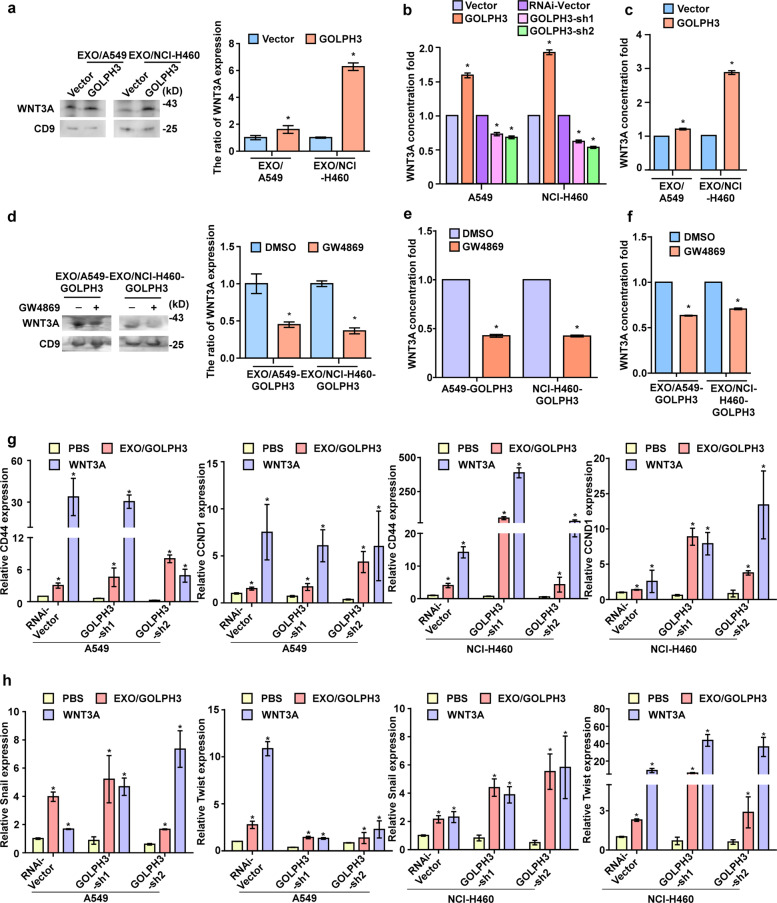
WNT3A in exosomes derived from GOLPH3-overexpressing cells enhances NSCLC cell metastasis and a stem cell-like phenotype. **a** Western blot analysis of WNT3A levels in exosomes derived from *GOLPH3*-overexpressing and vector control cells (left); quantification of WNT3A expression (right). **b** ELISA of WNT3A levels in *GOLPH3*-overexpressing, *GOLPH3*-silenced, or vector control cells. **c** ELISA of WNT3A levels in exosomes derived from *GOLPH3*-overexpressing and vector control cells. **d** Western blot analysis of WNT3A levels in exosomes derived from GOLPH3-overexpressing cells treated with GW4869 (left); quantification of WNT3A expression (right). **e** ELISA of WNT3A levels in *GOLPH3*-overexpressing cells treated with GW4869. **f** ELISA of WNT3A levels in exosomes of *GOLPH3*-overexpressing cells treated with GW4869 inhibitors. **g**, **h** Real-time PCR analysis of the mRNA expression stemness-related markers (**g**) and metastasis-related markers (**h**) in *GOLPH3*-silenced and vector control cells treated with exosomes or recombinant WNT3A protein.

Active WNT proteins are secreted in exosomes [14]. Based on previous finding that GOLPH3 regulates the protein trafficking of the WNT ligand secretion mediator (WLS) [21], we hypothesized that GOLPH3 regulates exosomal WNT protein secretion. To determine whether and which exosome-secreted WNT proteins are regulated by GOLPH3, we screened the literature and found that among the 19 WNT subfamily members, WNT1, WNT2, WNT3, WNT3A, WNT5A, WNT7B, and WNT11 were reported to be expressed in NSCLC [22, 23]. Western blot analysis showed that exosomes secreted by GOLPH3-overexpressing cells contained higher levels of WNT3A than control cells (Fig. 6a and Supplementary Fig. 6a). Furthermore, enzyme-linked immunosorbent assay (ELISA) analysis showed that the concentration of WNT3A in the culture medium increased after *GOLPH3* upregulation, whereas the concentration of WNT3A in the culture medium decreased after *GOLPH3* silencing (Fig. 6b). ELISA analysis also revealed increased WNT3A levels in exosomes secreted by *GOLPH3*-overexpressing cells (Fig. 6c). Western blot analysis showed that the levels of WNT3A in exosomes secreted by A549- and H460-GOLPH3 cells treated with GW4869 were lower than those in exosomes secreted by the control cells treated in the same way (Fig. 6d). ELISA analysis revealed that WNT3A levels were reduced in both the culture medium and exosomes of cells treated with GW4869 (Fig. 6e, f). In addition, real-time PCR analysis showed that metastasis and the stem cell-like phenotype of GOLPH3-silenced cells were rescued by treatment with these exosomes or exogenous WNT3A (Fig. 6g, h). Taken together, our results indicated that GOLPH3 promotes the secretion of exosomal WNT3A to enhance metastasis and the cancer stem cell-like phenotype of NSCLC cells.

### GOLPH3 interacts with CKAP4 to promote metastasis and the cancer stem cell-like phenotype of NSCLC

**Fig. 7:**
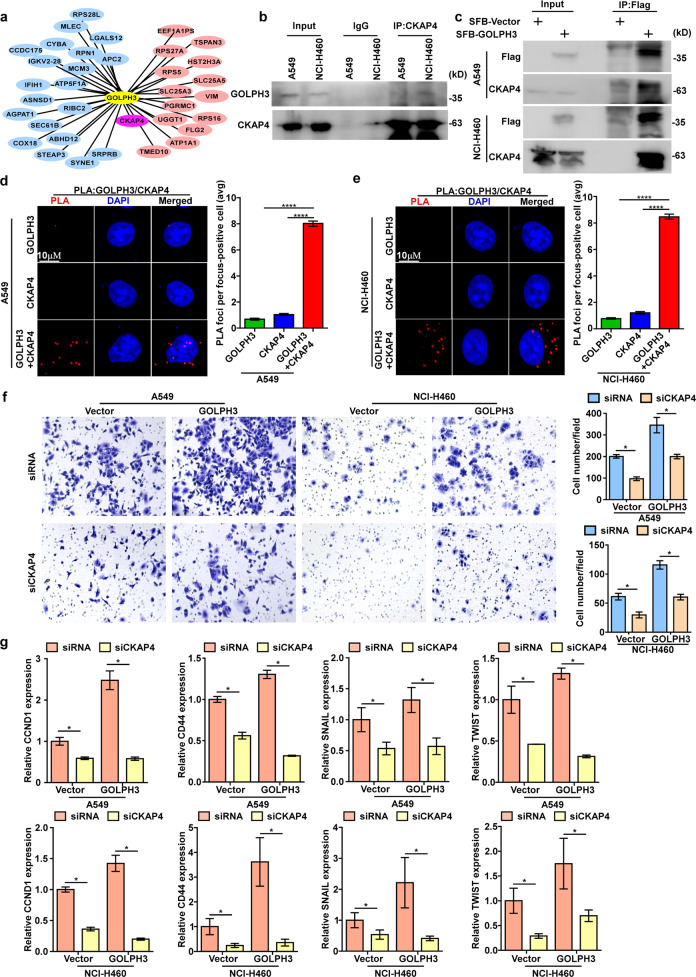
GOLPH3 interacts with CKAP4 to promote NSCLC metastasis and a cancer stem cell-like phenotype. **a** IP-MASS analysis showing GOLPH3-binding proteins. CKAP4 is the top differentially interacting protein in comparisons of SFB-vector and SFB-GOLPH3 cells by IP-MASS analysis; exosome-related proteins are marked in pink; the others are marked in blue. **b** Co-IP analysis of the interactions between GOLPH3 and CKAP4 in A549 and NCI-H460 cells. **c** Co-IP analysis of the interaction between GOLPH3 and CKAP4 in A549 and NCI-H460 cells transfected with SFB-Vector and SFB-GOLPH3. **d**, **e** Proximity ligation assay (PLA) of A549 (**d**) and NCI-H460 (**e**) cells with anti-GOLPH3 (Mx) and anti-CKAP4 (Rx) antibodies. Representative image of PLA signals (left); statistical analysis (right). Data represent the mean ± SEM of three independent experiments; ****P < 0.0001, one-way ANOVA. **f** Migration assay of *GOLPH3-*overexpressing cell lines treated with siCKAP4 or siRNA-vector (left). The number of cells that migrated to the lower Transwell chamber (right); *P < 0.05. **g** Real-time PCR analysis of the mRNA expression of metastasis-related or stemness-related proteins in *GOLPH3*-overexpressing and vector control cells with siCKAP4 or siRNA-vector treatment, respectively.

Next, we explored the mechanism by which GOLPH3 enhances the secretion of exosomal WNT3A. IP-MASS analysis showed that CKAP4 was the top differentially interacting protein in comparisons of SFB-vector and SFB-GOLPH3 cells (Fig. 7a). Co-immunoprecipitation (Co-IP) assays showed that endogenous GOLPH3 binds to CKAP4 in A549 cells and NCI-H460 cells (Fig. 7b). Co-IP analysis of A549 and NCI-H460 cells transfected with the SFB-Vector and SFB-GOLPH3 confirmed that GOLPH3 binds CKAP4 directly (Fig. 7c). As expected, we detected a physical interaction between GOLPH3 and CKAP4 (both endogenous and exogenous) by proximity ligation assay (PLA). Our data provided further evidence in support of our conclusion that GOLPH3 interacts with CKAP4 (Fig. 7d, e and Supplementary Fig. 7a, b). To investigate the function of the interaction between CKAP4 and GOLPH3 in NSCLC, we also used a CKAP4-siRNA to knock down the expression of CKAP4 in GOLPH3-overexpressing cells. Transwell assays without Matrigel (Fig. 7f) and real-time PCR analysis (Fig. 7g) showed that the stem cell-like phenotype and metastasis were decreased in GOLPH3-overexpressing cells with silenced CKAP4 expression. These data indicated that GOLPH3 interacts with CKAP4 to enhance NSCLC metastasis and the cancer stem cell-like phenotype.

### GOLPH3 increases CKAP4 levels in exosomes to promote the secretion of exosomal WNT3A

**Fig. 8:**
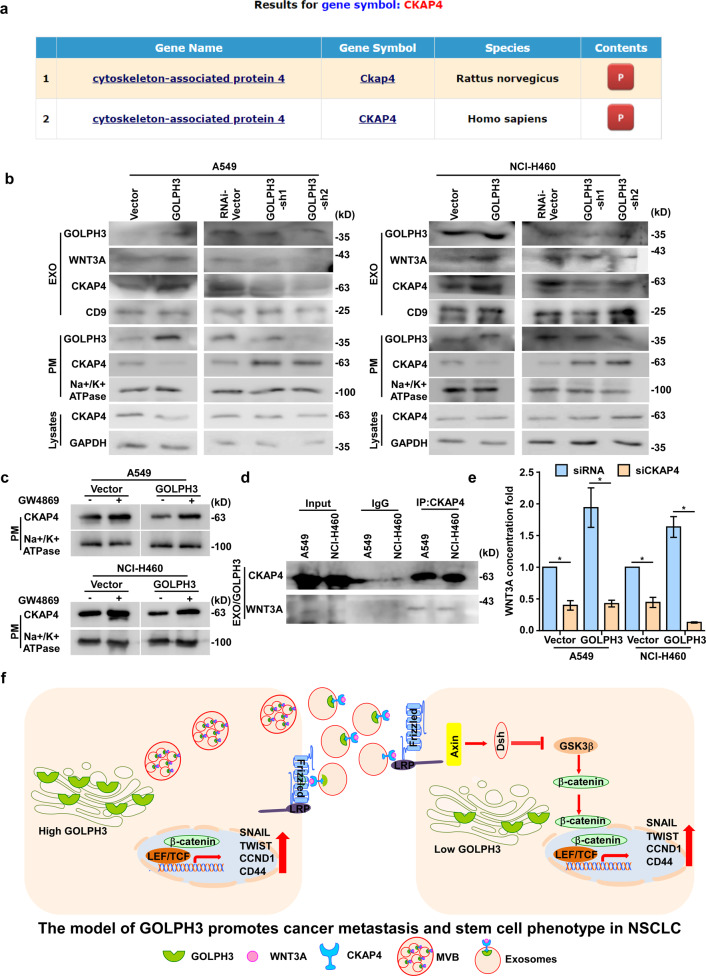
GOLPH3 enhances the secretion of CKAP4-containing exosomes to induce NSCLC metastasis and a cancer stem cell-like phenotype. **a** Query results for gene symbol *CKAP4* in ExoCarta. **b** Western blot analysis of GOLPH3, WNT3A, and CKAP4 levels in exosomes and CKAP4 levels in the plasma membrane (PM) or total lysate of *GOLPH3*-overexpressing or *GOLPH3*-silenced cells. **c** Western blot analysis of CKAP4 levels in the PM of *GOLPH3*-overexpressing or control cells treated with or without GW4869. **d** Co-IP analysis of the interactions between CKAP4 and WNT3A in exosomes derived from *GOLPH3*-overexpressing cells. **e** ELISA of WNT3A levels in *GOLPH3*-overexpressing and vector control cells treated with siCKAP4 or siRNA-vector. **f** Model of the mechanism by which *GOLPH3* overexpression promotes the secretion of CKAP4-containing exosomes and the transfer of WNT3A, to induce activation of the WNT signaling pathway and thereby, enhance metastasis and a stem cell-like phenotype in NSCLC.

Analysis the ExoCarta exosome database revealed that CKAP4 is also located in exosomes (Fig. 8a). We also found that CKAP4 levels in exosomes were increased by GOLPH3 overexpression in A549 and NCI-H460 cells (Fig. 8b and Supplementary Fig. 8a–d). Furthermore, the amount of plasma membrane (PM)-localized CKAP4 decreased as CKAP4 levels in exosomes increased (Fig. 8b and Supplementary Fig. 8b, d, e–h), although there was no significant change in the levels of total CKAP4 protein in total cell lysates (Fig. 8b and Supplementary Fig. 8i, j). Western blot analysis showed that CKAP4 accumulated in the PM when GOLPH3-overexpressing and vector control cells were treated with GW4869 (Fig. 8c), suggesting that GOLPH3 interacts with CKAP4 to enhance the formation of CKAP4-containing exosomes. Western blot analysis also showed that WNT3A levels in exosomes were increased in GOLPH3-overexpressing cells (Fig. 8b and Supplementary Fig. 8k, l). Co-IP assays showed that CKAP4 binds WNT3A in exosomes (Fig. 8d). ELISA analysis showed that WNT3A protein levels were also decreased in the culture medium of CKAP4-silenced *GOLPH*3-overexpressing cells (Fig. 8e). These results indicated that CKAP4-containing exosomes function as carriers to transfer WNT3A. Next, we treated *GOLPH3*-silenced cells with exosomes derived from *GOLPH*3-overexpressing cells transfected with CKAP4-siRNA or vector-siRNA. Real-time PCR analysis showed that exosomes from *GOLPH3*-overexpressing cells transfected with CKAP4-siRNA did not rescue cell metastasis and the stem cell-like phenotype of *GOLPH3*-silenced cells (Supplementary Fig. 9a, b). Collectively, these data provided evidence that GOLPH3 interacts with CKAP4 to enhance the secretion of exosomal WNT3A, thereby inducing metastasis and the cancer stem cell-like phenotype in NSCLC.

## Discussion

Previous studies have demonstrated the proliferative and anti-apoptotic effects of GOLPH3 as well as its role in efficient Golgi-to-PM trafficking in cancer [24–27]. In the present study, we evaluated the roles of GOLPH3 in cancer metastasis and exosomes. We revealed high *GOLPH3* expression in NSCLC tissues, which correlated positively with both poor prognosis and distal organ metastasis in patients with NSCLC. Mechanistically, we showed that *GOLPH3* interacts with CKAP4 to promote the formation of CKAP4-containing exosomes. Furthermore, we demonstrated that CKAP4 enhances the secretion of exosomal WNT3A by binding WNT3A in exosomes, which subsequently activates the WNT/β‑catenin pathway and leads to exosome-mediated distal metastasis.

Multiple lines of evidence show that exosomes bud from the endosome and PM [28, 29]. The multisubunit endosomal-sorting complex required for transport (ESCRT) machinery is a key regulator of exosome biogenesis [30]. The FYVE zinc-finger domain of the HRS subunit binds directly to the endosomal lipid phosphatidylinositol 3-phosphate (PtdIns(3)P) to induce ESCRT recruitment to endosomes [31, 32]. PtdIns(3)P-bound HRS recruits the ESCRT-I complex by binding to TSG101 via its carboxy-terminal domain. VPS28 then interacts with the stable heterotetrametric complex ESCRT-II by binding to the EAP45 subunit. Finally, VPS4 aids ESCRT-III disassembly and vesicle budding [30, 33]. GOLPH3 has been reported to interact with PI4P and MYO18A to participate in vesicle bud formation. In this study, we showed that exosomal CKAP4 levels were increased by overexpression of GOLPH3 in NSCLC cells and decreased by GOLPH3 knockdown. PM-localized CKAP4 levels were inversely proportional to the levels of exosomal CKAP4. Therefore, we speculated that GOLPH3 participates in the ESCRT pathway to enhance the formation of CKAP4-containing exosomes.

Previous studies have suggested that exosomes play key roles in tumor metastasis and stem cell-like phenotype in cancer cells [34–39]. For example, Chen et al. demonstrated that fibroblasts enhance breast cancer cell metastasis through paracrine transport of exosomal WNT10B [35]. Takizawa et al. found that chorionic villous trophoblast exosomal miR-520c-3p regulates cell invasion by targeting CD44 in extravillous trophoblasts [37]. Li et al. reported that cancer-secreted exosomal miR-21-5p promoted angiogenesis and vascular permeability by regulating the expression level of CCND1 in colorectal cancer [39]. In this study, we found that the GOLPH3-transduced cell exosomes treatment enhanced the cancer stem cell-like phenotype and cancer metastasis more effectively than treatment with recombinant WNT3A. Based on our results, we speculated that exosomes regulate the expression of stem cell-related markers mainly through activation of the Wnt/β-catenin signaling pathway, although other mechanisms might also be involved.

CKAP4 is a type II transmembrane protein originally located in the endoplasmic reticulum (ER) and binds to microtubules to organize the overall ER structure [40]. CKAP4, which functions as a receptor of various ligands, is detected on the cell surface membrane of lung cancer cells [41]. For instance, CKAP4 is a receptor for Dickkopf-1 (DKK1) proteins and is also a potential molecular marker of pancreatic cancer [42]. Kimura et al. found that the CRD1 domain of DKK1 binds directly to CKAP4, while the CRD2 domain of DKK1 plays a role in LRP6-binding and WNT inhibitory activities [43–46]. Interestingly, we found that the levels of both CKAP4 and WNT3A in exosomes were higher in *GOLPH*3-overexpressing cells compared to those in control cells. Co-IP assays showed that CKAP4 binds WNT3A in exosomes, although the potential role of WNT3A as an additional ligand for CKAP4 requires further investigation. Moreover, our results also showed that NSCLC cells expressing upregulated levels of GOLPH3 are more prone to intestinal metastasis. Genecards analysis revealed high expression of CKAP4 in the intestines, which might indicate intestinal tropism of NSCLC metastasis.

In conclusion, our results indicate that GOLPH3 enhances the secretion of exosomes containing CKAP4 and WNT3A, which activate the WNT/β-catenin pathway, thereby contributing to metastasis and the cancer stem cell-like phenotype in NSCLC (Fig. 8f). We also established a positive correlation between GOLPH3 expression and NSCLC metastasis, thus implicating GOLPH3 as a novel prognostic biomarker or potential therapeutic target for metastasis of NSCLC.

## Materials and methods

### Cell lines

Primary normal lung epithelial cells (NLEC) were provided by Professor Li Jun (Zhongshan School of Medicine, Sun Yat-sen University, China). 293T cells were provided by Professor Xingzhi Xu (Health Science Center of Shenzhen University, China). 293FT cells and lung cancer cell lines (A549, NCI-H1650, NCI-H358, NCI-H596, PAa, NCI-H460, and NCI-H292) were purchased from the American Type Culture Collection (ATCC, Manassas, VA, USA). NLEC cells were maintained in keratinocyte serum-free medium (Invitrogen, Carlsbad, CA, USA) with the addition of 1 mL defined keratinocyte-SFM growth supplement to 500 mL defined keratinocyte-SFM basal medium before use. 293T, 293FT, and lung cancer cells were maintained in Dulbecco’s modified Eagle’s medium (DMEM) supplemented with 10% fetal bovine serum (FBS). GW4869 (15 µM) was purchased from MedChemExpress (New Jersey, USA).

### Patient information and tissue specimens

This study was conducted using 132 primary paraffin-embedded, archived NSCLC specimens collected from patients diagnosed histopathologically and clinically at the Shenzhen People’s Hospital (China). Clinical and clinicopathological classification and staging were determined according to the Union for International Cancer Control (UICC)/American Joint Committee on Cancer (AJCC) TNM classification [46]. This study was approved by the Institutional Research Ethics Committee of Shenzhen People’s Hospital and consent was obtained from patients prior to inclusion of the clinical materials in this study.

### Immunohistochemistry (IHC) assay

IHC analysis was performed according to the IHC analysis kit instruction (ZSGB-BIO, Beijing, China). Briefly, paraffin-embedded NSCLC specimen sections (thickness, 4 µm) were deparaffinized with xylene, and then dehydrated using a graded series of ethanol solutions (100%, 95%, and 70%). Next, sections were submerged in EDTA-containing antigen retrieval buffer and microwaved for antigen retrieval. Anti-GOLPH3 (1:200; ABclonal, Wuhan, China; Cat. No. A13121) and anti-MUC1 (1:200; ABclonal, Wuhan, China; Cat. No. A0333) antibodies were used for IHC analysis of paraffin-embedded specimens of NSCLC patient tissues, mouse tumors tissues, and experimental lung metastatic lesion tissues. We assessed the expression of the indicated proteins in lesion tissues according to the staining index (SI), which was calculated as the staining intensity × the percentage of positive tumor cells. Staining intensity was scored as follows: 1, no staining; 2, weak staining (light yellow); 3, moderate staining (yellow-brown); and 4, strong staining (brown). The percentage of positive tumor cells was graded as follows: 0, no positive tumor cells; 1, <2% positive tumor cells; 2, 2–8% positive tumor cells; 3, 8–20% positive tumor cells; and 4, >20% positive tumor cells. Thus, specimens were assigned possible SI scores of 0, 1, 2, 3, 4, 6, 8, 9, 12, and 16. SI ≥ 8 was considered to represent high expression and cut-off values were determined based on a measure of heterogeneity using the log-rank test with respect to overall survival.

### RNA extraction and quantitative real-time PCR

Total RNA was extracted from NSCLC cells using RNAiso Plus kits (9109, TaKaRa, Shiga, Japan). Quantitative real-time PCR (qRT-PCR) analysis was performed using SYBR Green (Nanjing Vazyme, China) with a CFX96 Real-Time PCR system (Bio-Rad, CA, USA). The expression data were normalized to the geometric mean of the expression of the housekeeping gene *GAPDH*. Relative gene expression was calculated using the 2^−ΔΔCt^ method. The primer sequences used for this analysis are listed in Supplemental Table 1.

### Plasmids, siRNAs, and transfection

The human *GOLPH3* cDNA was amplified by PCR and cloned into the pMSCV-puro vector. The primer sequences used for this analysis are listed in Supplemental Table 2. Two human *GOLPH3*-targeting short hairpin RNA (shRNA) sequences (shRNA#1: GCATGTTAAGGAAACTCAGCC; shRNA#2: GCAGCGCCTCATCAAGAAAGT) were cloned into a SUPER.retro.puro vector (OligoEngine, Seattle, WA, USA) to knockdown *GOLPH3* expression. CKAP4-siRNA (5′-TCAGCGAAGTGCTGCAGAA-3′) was purchased from Ruibo Biotechnologies (Guangzhou, China). Stable NSCLC cell lines were generated by retroviral infection and selected for 10 days using 0.5 μg/ml puromycin (Solarbio, Beijing, China. Cat. No. P8230), as described previously [9].

### Western blot analysis

Western blot analysis was conducted as described previously [9]. Briefly, protein concentration was quantified using a Pierce BCA Protein Assay kit (Thermo Fisher Scientific, Waltham, MA, USA). Equal amounts of protein were separated by sodium dodecyl sulfate-polyacrylamide gel electrophoresis (SDS-PAGE). The following primary antibodies were used: anti-GOLPH3 (1:1000, Proteintech, Rosemont, IL, USA; Cat. No. 19112-1-AP), anti-β-catenin (1:5000, Pharmingen/BD Biosciences, Bedford, MA, USA; Cat. No. 610254), anti-WNT3A (1:1000, Cell Signaling Technology, Danvers, MA, USA; Cat. No. 2721S), anti-WNT7B (1:1000, Bioworld Technology, St. Louis Park, MN, USA; Cat. No. BS71443), anti-WNT1 (1:1000, ABclonal, Wuhan, China; Cat. No. A2475), anti-WNT11 (1:2000, Abcepta, San Diego, CA, USA; Cat. No. 096014), anti-WNT2 (1:1000, Bioworld Technology; Cat. No. BS7909), anti-WNT3 (1:500, ABclonal; Cat. No. A9328), anti-WNT5A (1:500, ABclonal; Cat. No. A19133), CD9 (1:1000, ZENBIO, Durham, NC, USA; Cat. No. 382758), anti-CD81 (1:1000, ZENBIO; Cat. No. 393866), anti-CKAP4 (1:500; Santa Cruz Biotechnology, Santa Cruz, CA, USA; Cat. No. sc-393544), anti-α-tubulin (1:5000, Abcam, Cambridge, MA, USA; Cat. No. Ab7291), and anti-histone 2A (H2A) (1:1000, Cell Signaling Technology, Cat. No. 12349S). Protein bands were quantified by Image J (Version 1.8.0, NIH, USA, https://imagej.nih.gov/ij/index.html). Primary antibodies against CD9, Na^+^/K^+^ ATPase, and GAPDH were used as protein loading controls.

### Transwell assay and Transwell matrix invasion assay

The upper chamber of the BioCoat™ Invasion Chambers (Corning Inc., Corning, NY, USA) was coated with prediluted Matrigel (Solarbio, Beijing, China; Cat. No. 8370) for the cell invasion assay, or without, for the cell migration assay. The 24-well plates with Chambers coated with Matrigel were then incubated at 37 °C for 30 min. A serum-free cell suspension was seeded onto the surface of the polycarbonate Transwell filter in 24-well plates (5 × 10^4^ cells/well). The lower chamber was filled with medium supplemented with 20% FBS, and the cells were cultured at 37 °C under 5% CO_2_ for 24 h. The cells remaining in the upper chamber were removed using cotton swabs and the cells that invaded the Matrigel and migrated to the lower side of the membrane were fixed with 1% paraformaldehyde for 10 min, stained with crystal violet for 5 min, and counted under a microscope.

### Wound-healing assay

For each group, cells were seeded into six-well plates (1 × 10^6^ cells/well). The cells were cultured in DMEM containing 10% FBS to 90% confluence. A wound (scratch) was then introduced into the cell layer using a sterile 200-μl pipette tip and the cells in suspension were washed off using the medium. The wound (scratch) gap was measured at 0, 12, 24, and 48 h.

### Sphere-formation assays

For each group, cells were seeded into ultra-low cluster six-well plates (500 cells/well) and cultured at 37 °C under 5% CO_2_ in DMEM/F12 serum-free medium supplemented with 2% B27 (Invitrogen, Waltham, MA, USA; Cat. No. 12587010), 20 ng/ml EGF (PeproTech, Rocky Hill, NJ, USA; Cat. No. 37000015), 20 ng/ml bFGF (Cat. No. 100-18B), 5 μg/ml insulin (PeproTech; Cat. No. 100-11), and 0.4% BSA (Sigma-Aldrich, St. Louis, MO, USA; Cat. No. A1933-1G). Cells were cultured for approximately 10 days before tumor spheres (tight, spherical, nonadherent masses >50 µm in diameter) were counted under an inverted microscope, and images were captured (CKX53, Olympus, Japan).

### Flow cytometry

Cells were trypsinized and suspended at 1 × 10^6^ cells/ml in Dulbecco’s modified Eagle’s medium supplemented with 2% FBS. Cells were then incubated with or without 100 μM verapamil (Sigma-Aldrich) at 37 °C for 30 min. The cell suspension was then treated with 5 μg/ml Hoechst 33342 (Sigma-Aldrich; Cat. No. B2261) for 90 min at 37 °C, and swirled every 10 min. After incubation on ice for 10 min, the cells were washed with ice-cold phosphate-buffered saline (PBS) and processed for flow cytometric analysis on a BD FACSCanto II flow cytometer (BD Biosciences, San Jose, CA, USA). Data were analyzed using FlowJo software (Tree Star Inc, Ashland, OR, USA).

### Tumor xenograft models, immunohistochemistry (IHC), and hematoxylin and eosin (H&E) staining

Tumor xenograft models were established in male BALB/c nude mice (aged 5–6 weeks, 16–18 g) purchased from Beijing Vital River Laboratory Animal Technology Co., Ltd. (China). All nude mice were housed in a barrier facility in the Peking University Laboratory Animal Center of Shenzhen Graduate School. For bioluminescence assays, A549 cells were stably transduced with pMSCV-luciferase-neo plasmid, followed by transduction with pMSCV-GOLPH3-puro, pSuper-GOLPH3-RNAi#2-puro or empty vector to generate the A549-luc-GOLPH3, A549-luc-RNAi-vector, A549-luc-vector, and A549-luc-GOLOH3-shRNA#2 cell lines. To establish distal metastasis animal models, nude mice (five mice/group) were injected with GOLPH3-overexpressing or GOLPH3-silenced cells or control cells (1 × 10^6^ cells) via the lateral tail vein or the left cardiac ventricle. Bioluminescence signals were monitored for each animal every 7 days for 30 days using the Xenogen IVIS Spectrum (Caliper Life Sciences). At the end of the experiment, systemic organs with metastatic signals were resected and stained with H&E for histological confirmation of metastasizing tumor cells. IHC staining was then conducted using anti-MUC1 antibodies (1:200, ABclonal Technology, Wuhan, China; Cat. No. A19081). For tumorigenesis experiments, A549-GOLPH3/vector cells or A549-GOLPH3-RNAi#2/vector cells (1 × 10^6^, 1 × 10^5^, 1 × 10^4^, or 1 × 10^3^ cells in 25% Matrigel [final concentration]) were injected into the left inguinal folds of nude mice. Tumors were measured using an external caliper every 3 days for 45 days and the volume was calculated using the following equation: (L × W^2^)/2. At the end of the experiment, the tumors and metastatic organs were excised for H&E staining and immunostaining using anti-GOLPH3 (1:200, ABclonal Technology, Wuhan, China; Cat. No. A13121), anti-CD133 (1:200, ABclonal Technology, Wuhan, China; Cat. No. A0219) and anti-ZEB1 (1:200, ABclonal Technology, Wuhan, China; Cat. No. A1500) antibodies.

### Luciferase-reporter assay

NSCLC cells were seeded into 24-well plates (3 × 10^4^ cells/well in triplicate) and cultured at 37 °C under 5% CO_2_ for 24 h. Cells were then transiently transfected with TOP flash or FOP flash and Renilla pRL-TK plasmids using Lipofectamine 3000 (Life Technologies, Carlsbad, CA, USA) according to the manufacturer’s protocol. After 48 h, luciferase activity was determined using a dual-luciferase reporter assay (Promega, Madison, WI, USA). Briefly, the culture medium was discarded, and the cells were washed with PBS and lysed with passive lysis buffer (Promega). The cell lysates were transferred to a 96-well plate luminometer (Corning, New York, USA) and analyzed using a microplate reader (Bioteck, Winooski, VT, USA, #Synergy H/MD).

### Nuclear extract preparation

NSCLC cells (2 × 10^6^ cells) were cultured at 37 °C under 5% CO_2_ in DMEM supplemented with 10% FBS in 10-cm dishes. After 24 h, NSCLC cells were washed twice with 5 ml ice-cold PBS containing phosphatase inhibitors (Cocktail, 1:100. Bimake, Houston, USA; Cat. No. B15001). Subsequently, 3 ml lysis buffer containing phosphatase inhibitors was added to the cells. The cells were then scraped from 10-cm dishes and transferred cells into a pre-chilled 15-ml conical tube. The samples were centrifuged at 200×*g* for 5 min at 4 °C for collection of the pellets containing nuclei. Nuclear proteins were isolated using a Nuclear Extract Kit (Active Motif, Rixensart, Belgium; Cat. No. 40010) for western blot analysis.

### Membrane protein extraction

NSCLC cells (5 × 10^7^ cells) were cultured at 37 °C under 5% CO_2_ in DMEM supplemented with 10% FBS in 15-cm dishes. At ~90% confluence, the cells were washed with PBS and scraped off the dishes. The cells were centrifuged 200×*g* for 5 min at 4 °C for collection of the cell pellet. The proteins in the cell membrane and cytosol were then isolated using a Membrane and Cytosol Protein Extraction Kit (Yeasen Biotech Co. Ltd., Shanghai, China; Cat. No. 20127ES50) according to the manufacturer’s instructions and analyzed by Western blotting and functional assays.

### Immunofluorescence assay

NSCLC cells (3 × 10^4^ cells/well) were plated on coverslips and cultured at 37 °C under 5% CO_2_ for 24 h before being washed twice with ice-cold PBS, and fixed with 4% paraformaldehyde for 15 min. The cells were then incubated with anti-β-catenin (1:100; Cell Signaling Technology, Boston, USA; Cat. No. 8480S) overnight at 4 °C. After being washed with PBS, the cells were incubated with fluorescein-conjugated goat anti-rabbit secondary antibodies (IgG, 1:500; Abcam, Cambridge, UK; Cat. No. Ab150077) at room temperature for 1 h. Cell nuclei were counterstained with DAPI (5 ng/ml) for 10 min. After being washed three times with PBS, the coverslips were mounted with an anti-fading reagent (Invitrogen) and evaluated at ×400 magnification using the FV10-ASW 4.2 computerized image analysis system (Olympus, Tokyo, Japan). The MOD of the nuclear β-catenin staining signals was determined using Image J software (Version 1.48, https://imagej.nih.gov/ij/). The MOD data were analyzed statistically using the t-test to compare the MOD differences between the groups; *P < 0.05.

### Co-immunoprecipitation (Co-IP) assay

293T, A549, and NCI-H460 cells were cultured in 100-mm dishes and transiently transfected with SFB-vector or SFB-GOLPH3 plasmids. At 80–90% confluence, the cells were washed three times with ice-cold PBS and lysed on ice for 30 min using immunoprecipitation (IP) lysis buffer. The lysates were clarified by centrifugation at 14,000×*g* for 10 min and incubated with 20 μl of agarose beads (20421, Thermo, Waltham, MA, USA) for 1 h at 4 °C with rotation. Following centrifugation at 2000×*g* for 1 min, the supernatants were collected and incubated overnight at 4 °C with 20 μl anti-FLAG antibody-cross-linked protein G-agarose beads. The samples were centrifuged at 2000×*g* for 10 min, and the agarose beads were washed six times with lysis buffer. The pelleted beads were resuspended in 30 μl loading buffer for SDS-PAGE followed by western blotting using the indicated antibodies.

### ELISA

WNT3A levels were measured using a WNT3A (CSB-EL026138) ELISA kit (Cusabio Houston TX, USA) according to the manufacturer’s instructions.

### Exosome isolation

The NSCLC cell lines were cultured in DMEM containing 10% exosome-depleted FBS. After 48 h, the medium was collected for exosome isolation. Briefly, the culture medium was collected into an ultrafiltration tube by centrifugation at 2000×*g* for 10 min. To purify exosomes from the medium, we used the ExoQuick-TC™ Exosome Precipitation Solution Kit (System Biosciences, Palo Alto, CA, USA) according to the manufacturer’s instructions. Subsequently, the exosome pellets were resuspended in PBS and filtered (0.22-μm pore size; Millipore, Billerica, MA, USA) for functional assays or protein detection. The size and quality of the exosomes were determined using an LM10-HS NanoSight instrument (Malvern Instruments, Malvern, UK) and NTA software (Malvern Instruments). The exosomes were examined using a transmission electron microscope (PHILIPS-TECNAI 10, Philips, Amsterdam, Netherlands) at 120 kV. For in vitro assays, purified exosomes were used at 20 µg/ml.

### Bioinformatic analysis

(GSEA) http://software.broadinstitute.org/gsea/msigdb/ in dex.jsp) software programs were used to analyze the Gene Expression Omnibus NSCLC dataset (GEO75037). We used DAVID Bioinformatics Resources 6.8, DAVID: Functional Annotation Tools, (https://david.ncifcrf.gov/tools.jsp) for GO analysis of IP-MASS-identified proteins. The exosome database ExoCarta (http://www.exocarta.org) was used to query CKAP4 expression in exosomes. The Genecards (http://www.genecards.org) was used to query CKAP4 expression in human organs.

### Proximity ligation assay (PLA)

A549 and NCI-H460 cells were seeded in six-well plates with slides (1.5 × 10^5^ cells/well). Cells were transfected with SFB-Vector or SFB-GOLPH3 and fixed with 4% paraformaldehyde (PFA) for 10 min. After washing with PBS, cells were permeabilized with 0.5% Triton X-100 for 3 min prior to blocking with 2% BSA (in PBS) for 0.5–1 h. Cells then were incubated with an appropriate concentration of primary antibody (mouse anti-FLAG: 1:100, Sigma, St. Louis, MO, USA; Cat. No. F1804; rabbit anti-CKAP4: 1:50, Novus, Colorado, USA; Cat. No. NBP1-26643; mouse anti-GOLPH3: 1:50, Proteintech, Rosemont, IL, USA; Cat. No. 67777-1-Ig) overnight at 4 °C. The PLA was performed using a Duolink in situ fluorescence kit (Sigma-Aldrich, St. Louis, MO, USA; Cat. No. DUO92101) according to the manufacturer’s instructions. Images were acquired using DragonFly confocal imaging system (Andor) and data were analyzed using Image J and GraphPad Prism.

### Statistical analysis

All statistical analyses were performed using SPSS version 21.0 (IBM Corp., Armonk, NY, USA) and GraphPad Prism (version 8). The two-tailed paired Student’s *t* test was used for comparisons of two groups. The *χ*^*2*^ test was used to assess the relationship between GOLPH3 expression and clinicopathological characteristics. The Kaplan–Meier method was used to plot the survival curves and the log-rank test was used to compare groups. Bivariate correlations between GOLPH3 expression and clinical stage, T, N, or M stage were determined using Spearman’s rank correlation analysis. *P* < 0.05 was considered to indicate statistical significance.

## Supplementary information

Supplementary figure legends

Supplementary figure 1

Supplementary figure 2

Supplementary figure 3

Supplementary figure 4

Supplementary figure 5

Supplementary figure 6

Supplementary figure 7

Supplementary figure 8

Supplementary figure 9

Supplementary table 1

Supplementary table 2

## Data Availability

All data generated or analyzed during this study are included in this published article and its supplementary information files. Other data supporting the results of this subject can be obtained from the corresponding author in accordance with reasonable regulations.
