# Supplementary material for: GOLPH3/CKAP4 promotes metastasis and tumorigenicity by enhancing the secretion of exosomal WNT3A in non-small-cell lung cancer
Source: Cell Death Dis. 2021 Oct 21;12(11):976. doi: 10.1038/s41419-021-04265-8 (PMC8528870; doi:10.1038/s41419-021-04265-8)
Supplement: Supplementary file 1 — Supplementary figure legends [file 41419_2021_4265_MOESM1_ESM.docx]

**Supplementary Fig. 1 Elevated GOLPH3 expression is associated with the clinicopathologic characteristics of NSCLC. (a-h)** Kaplan–Meier analysis of overall survival in subgroups of clinical stage I+II **(a)**, III+IV **(b)**, T classification **(c, d)**, N classification **(e, f)**, and M classification **(g, h)**. *P-*values determined by log-rank test.

**Supplementary Fig. 2 GOLPH3 promotes NSCLC cell metastasis and a stem cell‑like phenotype. (a)** Wound healing assay of cell migration ability of the indicated cell lines stably expressing *GOLPH3* (left). Quantification of the wound healing assay data (right); **P* < 0.05. **(b)** Real-time PCR analysis of the mRNA expression of stemness-related markers in the indicated NSCLC cells. **(c)** Effect of GOLPH3 on the tumorigenicity of NSCLC cells *in vivo* (n = 9/group).

**Supplementary Fig. 3 *GOLPH3* overexpression enhances activation of the WNT/β-catenin signaling pathway. (a)** Western blot analysis of β-catenin expression in the nuclear fractions of the indicated cells. **(b, c)** Representative images of immunofluorescence staining in A549 **(b)** or NCI-H460 **(c)** stable cell lines (left). Quantification of nuclear β-catenin expression (right). **(d, e)** Real-time PCR analysis of the mRNA expression of WNT/β-catenin target genes. Data represent the mean ± SD of three independent experiments; **P* < 0.05.

**Supplementary Fig. 4 GOLPH3 regulates exosomal WNT3A secretion** **in NSCLC.** **(a-d)** Nanoparticle Tracking Analysis (NTA) of the size of the exosomes (approximately 100 nm) with no difference between those derived from *GOLPH3* overexpressing and vector control cells. Peak analysis of exosomes secreted by *GOLPH3* overexpressing and vector cells is shown.

**Supplementary Fig. 5 Exosomes derived from *GOLPH3*-overexpressing cells regulate NSCLC stem cell-like phenotype through activation of the WNT/β-catenin signaling pathway. (a)** Real-time PCR analysis of the mRNA expression of stemness-related markers in *GOLPH3*-overexpressing and vector cells treated with GW4869.

**Supplementary Fig. 6 WNT3A in exosomes derived from *GOLPH3*-overexpressing cells enhances NSCLC cell metastasis and a stem cell-like phenotype. (a)** Western blot analysis of WNT ligand levels in exosomes derived from *GOLPH3*-overexpressing and vector control cells.

**Supplementary Fig. 7 GOLPH3 interacts with CKAP4. (a)** Western blot confirmation of A549 and NCI-H460 cell transfection with SFB-Vector and SFB-GOLPH3 plasmids. **(b)** PLA analysis of A549 and NCI-H460 cells transfected with SFB-Vector and SFB-GOLPH3 plasmids using anti-FLAG (Mx) and anti-CKAP4 (Rx) antibodies. Representative image of PLA signals (left); statistical analysis (right). Data represent the mean ± SEM of three independent experiments. *****P* < 0.0001, one-way ANOVA.

**Supplementary Fig. 8 Quantification of Western blots shown in Figure 8b. (a-d)** Quantification of GOLPH3 **(a, c)** and CKAP4 **(b, d)** expression in exosomes derived from stably transfected A549 **(a, b)** and NCI-H460 **(c, d)** cell lines. **(e-h)** Quantification of GOLPH3**(e, g)** and CKAP4 **(f, h)** expression in PM of stably transfected A549 **(e, f)** and NCI-H460 **(g, h)** cell lines. **(i, j)** Quantification of CKAP4 expression in stably transfected A549 **(i)** and NCI-H460 **(j)**cell lines. **(k, l)** Quantification of WNT3A expression in exosomes derived from stably transfected A549(k) and NCI-H460 (l) cell lines.

**Supplementary Fig. 9 GOLPH3** **promotes the secretion of exosomes containing CKAP4-WNT3A, inducing NSCLC metastasis and a cancer stem cell-like phenotype. (a, b)** Real-time PCR analysis of the mRNA expression of metastasis-related **(a)** and stemness-related **(b)** markers in *GOLPH3*-silenced cells with exosomes derived from the *GOLPH3*-overexpressing cells treated with CKAP4-siRNA or vector control.
