# Supplementary figures and images for: GOLPH3/CKAP4 promotes metastasis and tumorigenicity by enhancing the secretion of exosomal WNT3A in non-small-cell lung cancer
Source: Cell Death Dis. 2021 Oct 21;12(11):976. doi: 10.1038/s41419-021-04265-8 (PMC8528870; doi:10.1038/s41419-021-04265-8)

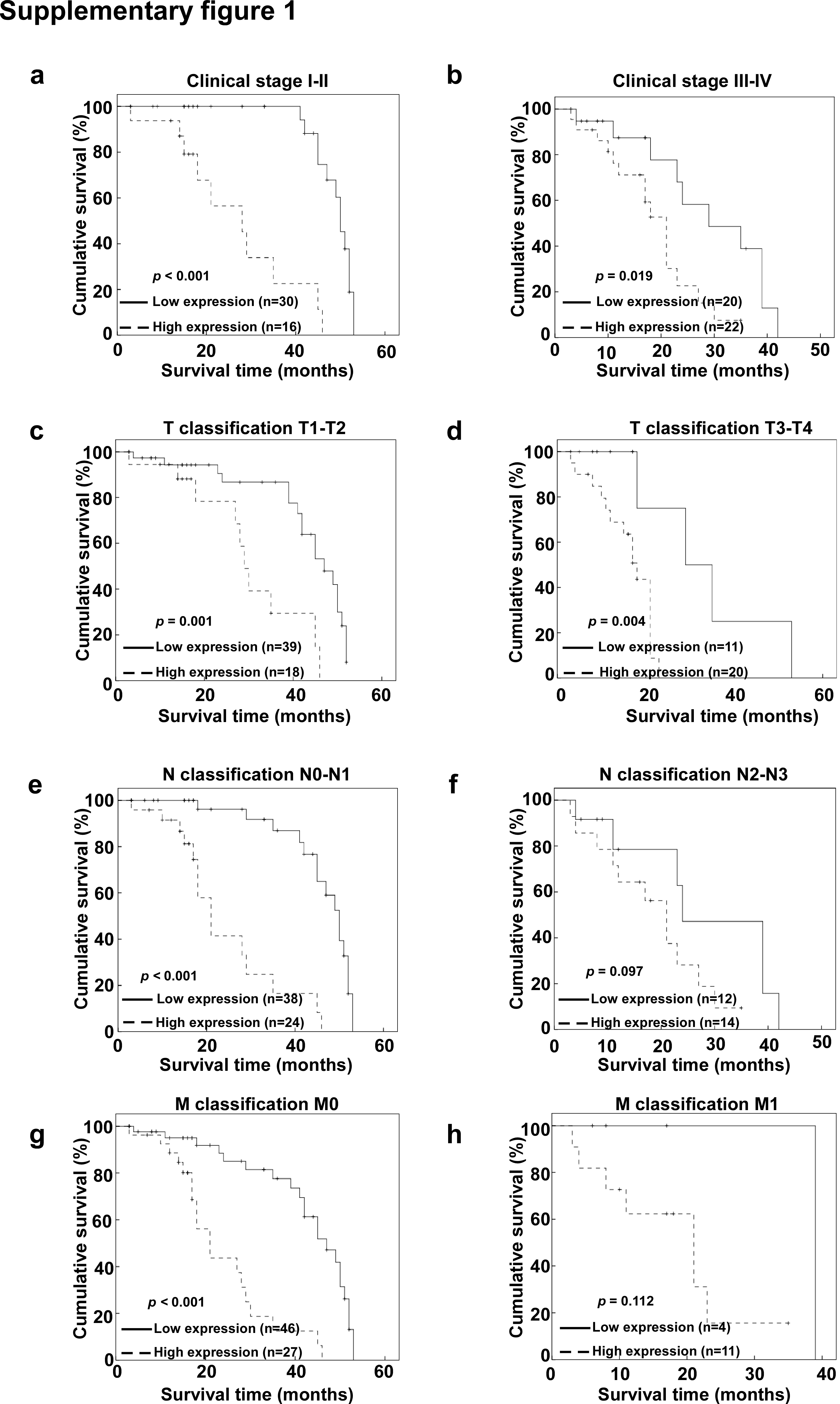

Supplement: Supplementary file 2 — Supplementary figure 1 [file 41419_2021_4265_MOESM2_ESM.tif]

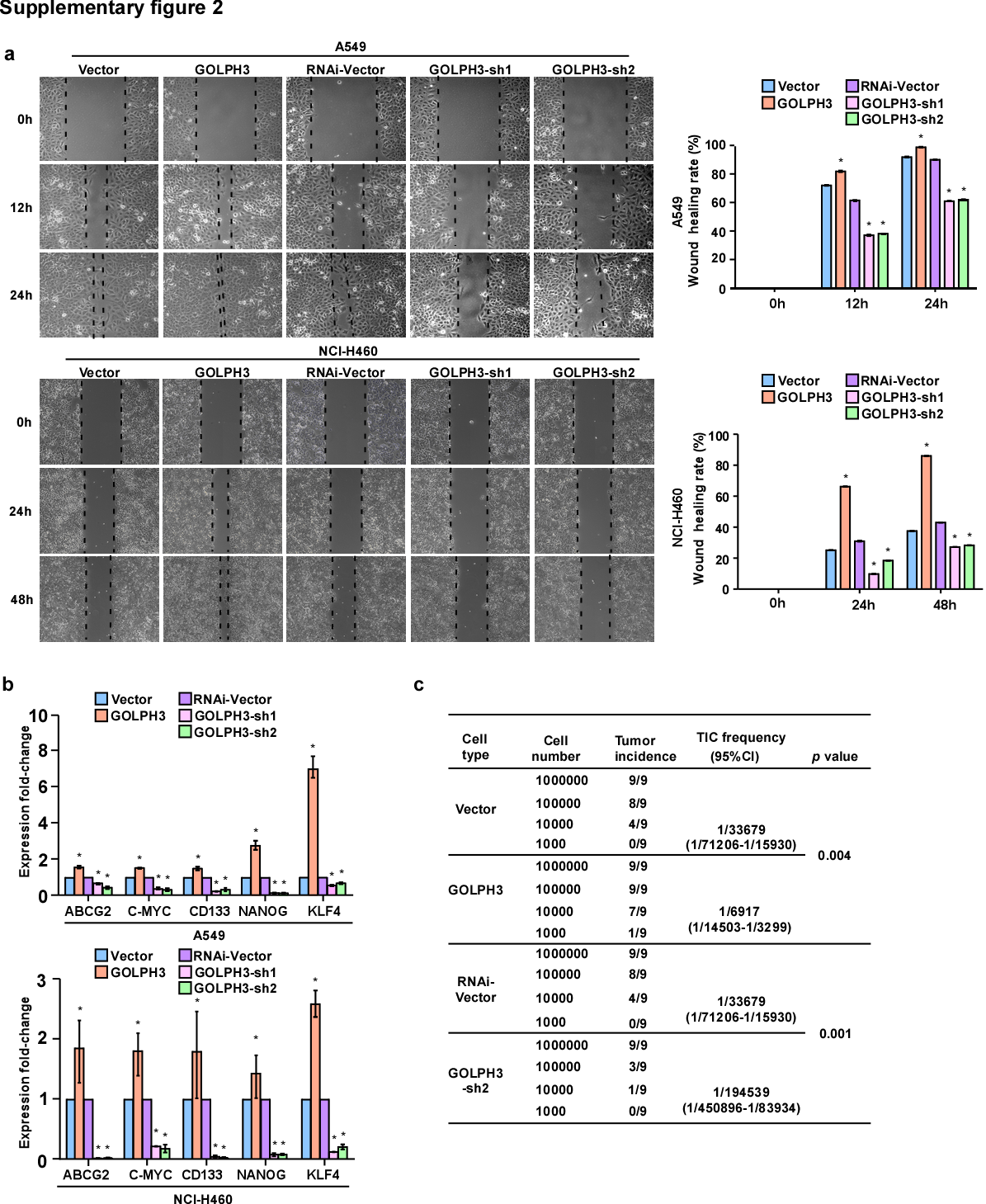

Supplement: Supplementary file 3 — Supplementary figure 2 [file 41419_2021_4265_MOESM3_ESM.tif]

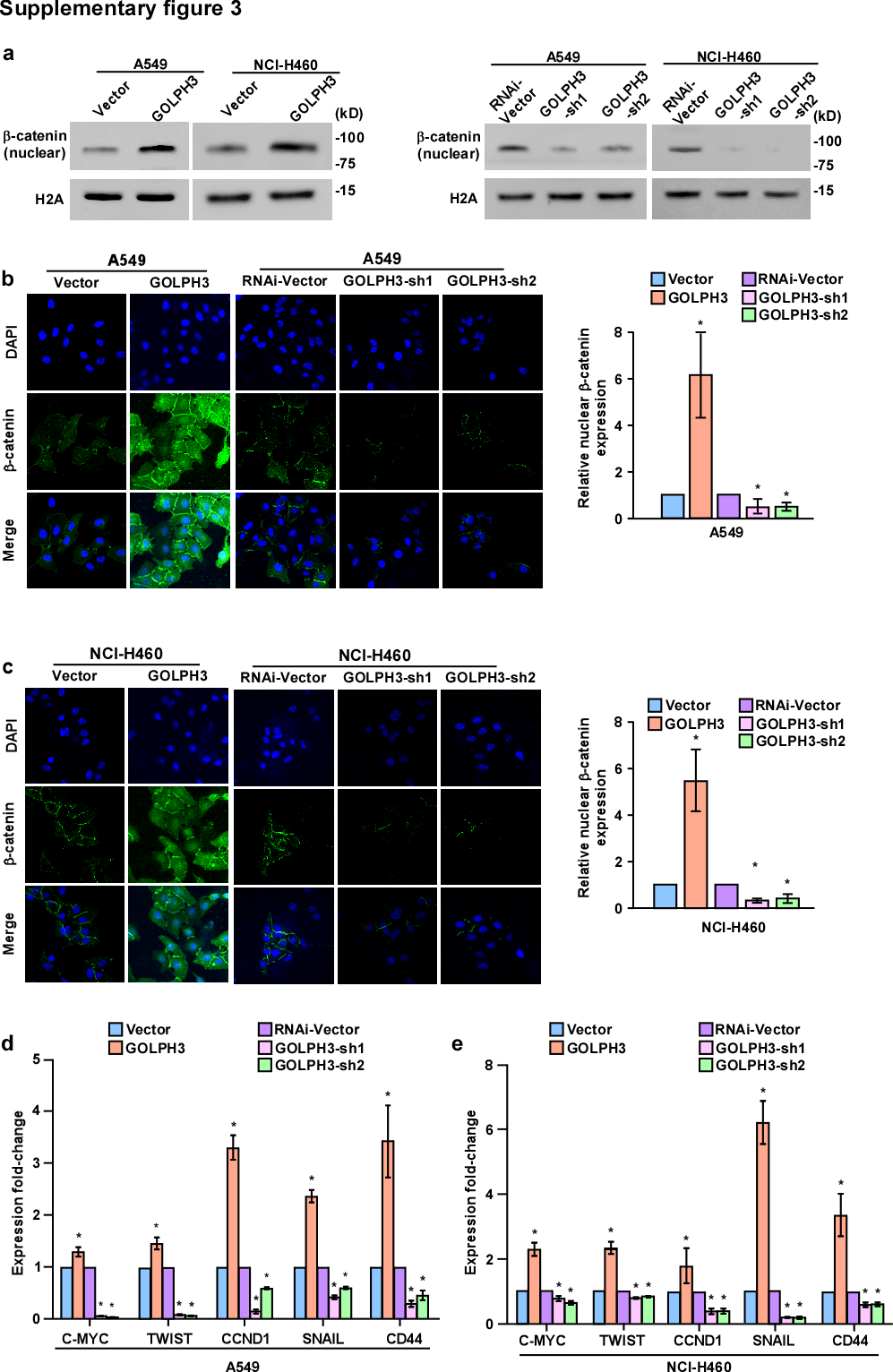

Supplement: Supplementary file 4 — Supplementary figure 3 [file 41419_2021_4265_MOESM4_ESM.tif]

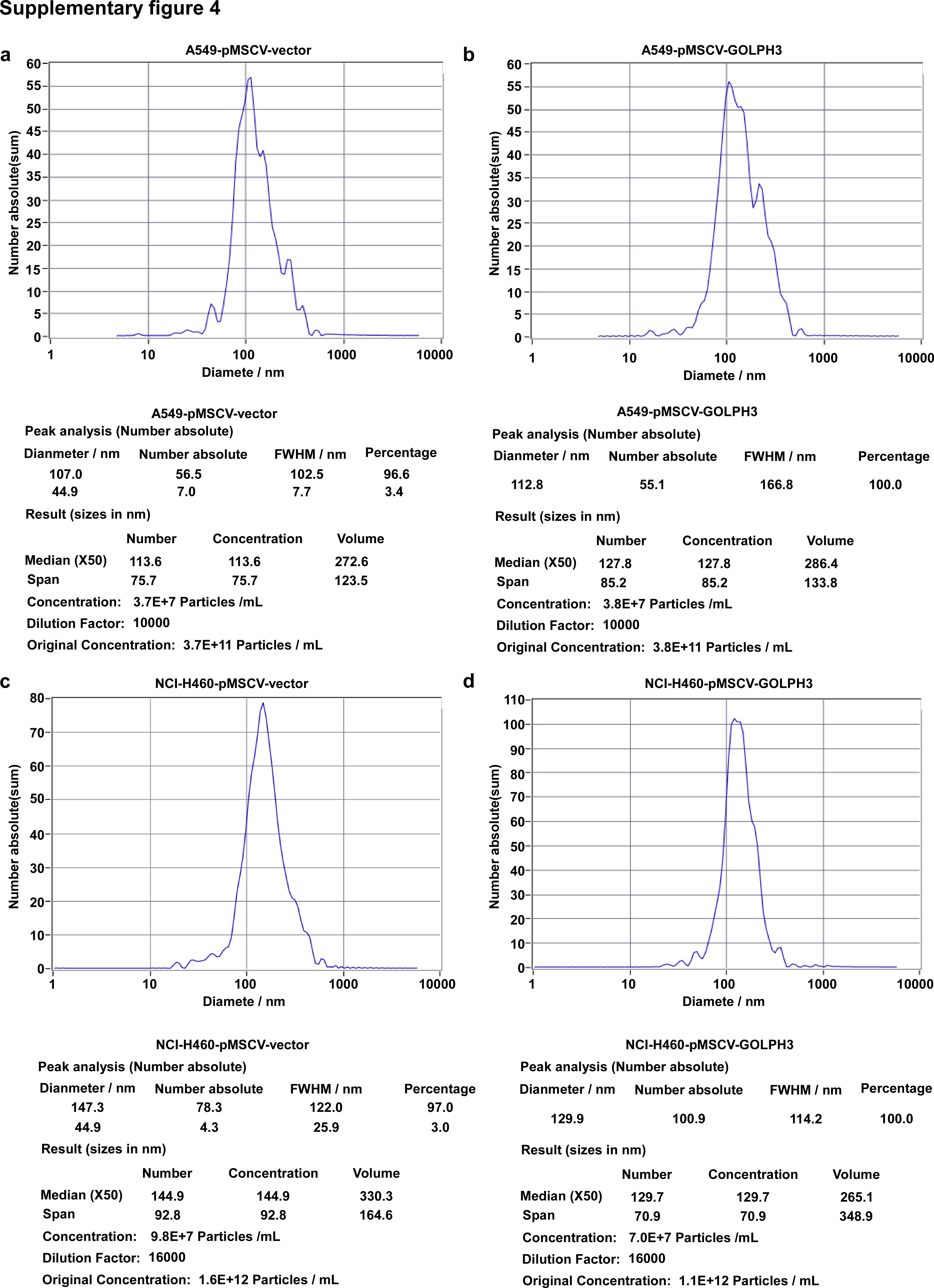

Supplement: Supplementary file 5 — Supplementary figure 4 [file 41419_2021_4265_MOESM5_ESM.tif]

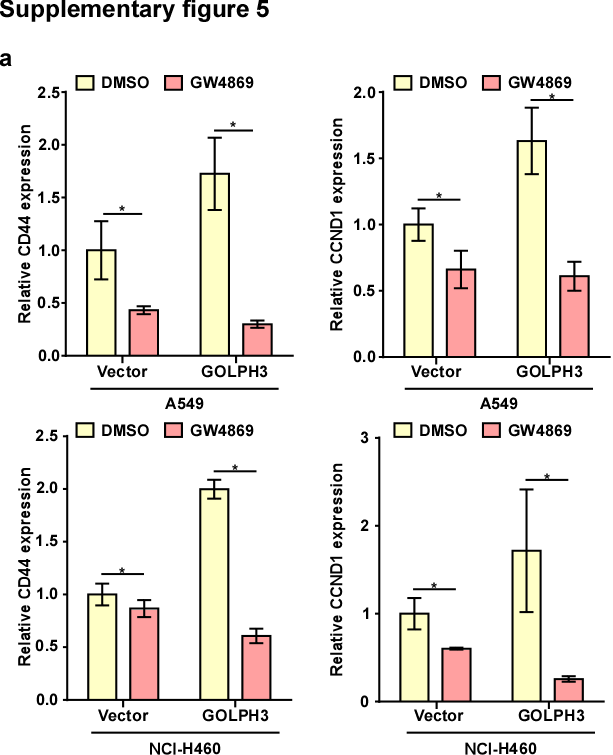

Supplement: Supplementary file 6 — Supplementary figure 5 [file 41419_2021_4265_MOESM6_ESM.tif]

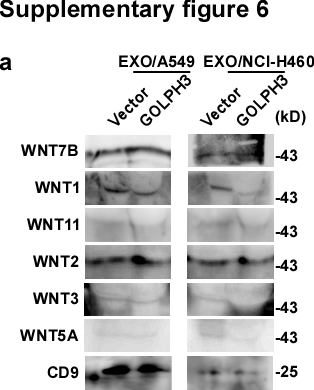

Supplement: Supplementary file 7 — Supplementary figure 6 [file 41419_2021_4265_MOESM7_ESM.tif]

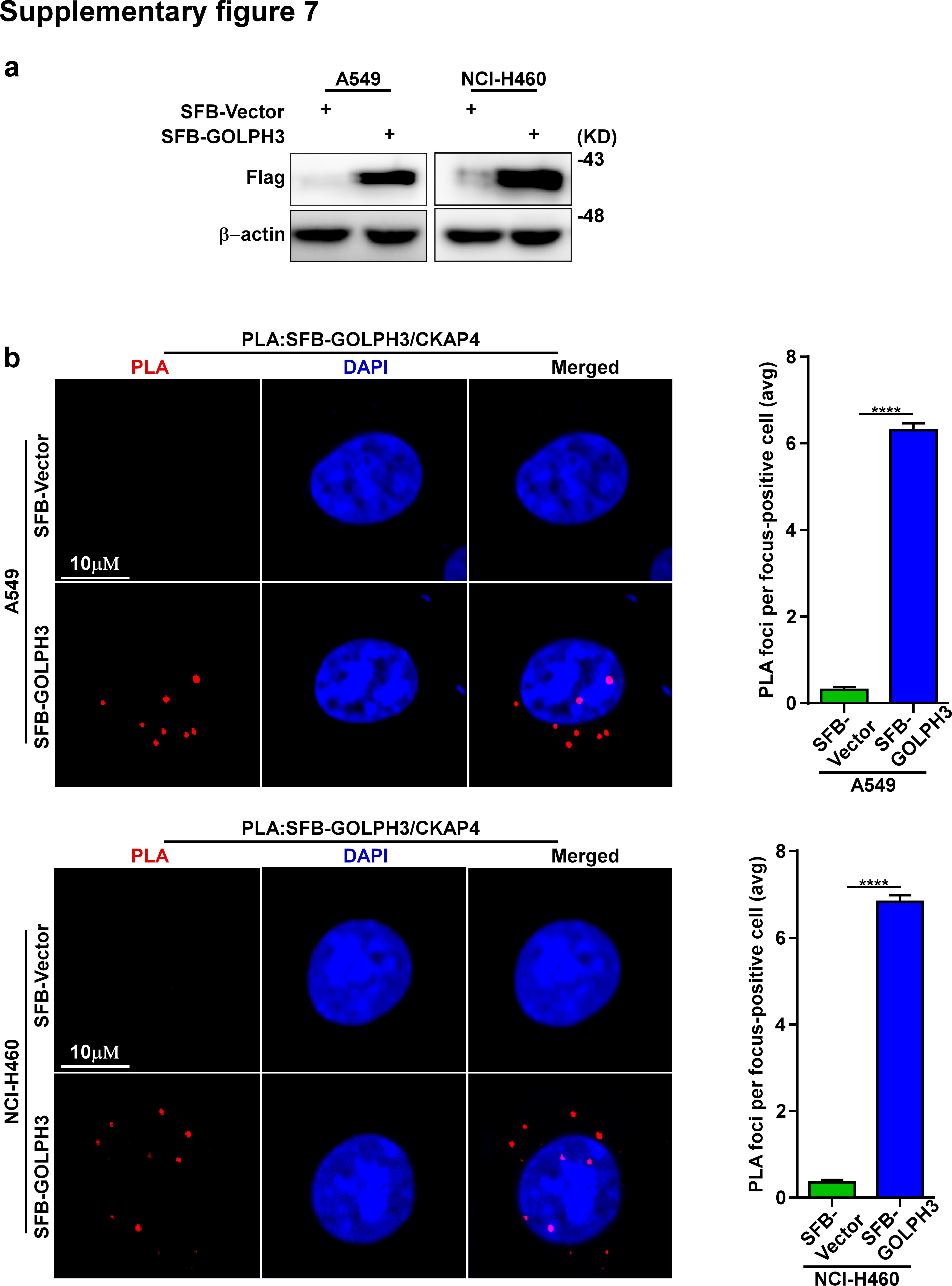

Supplement: Supplementary file 8 — Supplementary figure 7 [file 41419_2021_4265_MOESM8_ESM.tif]

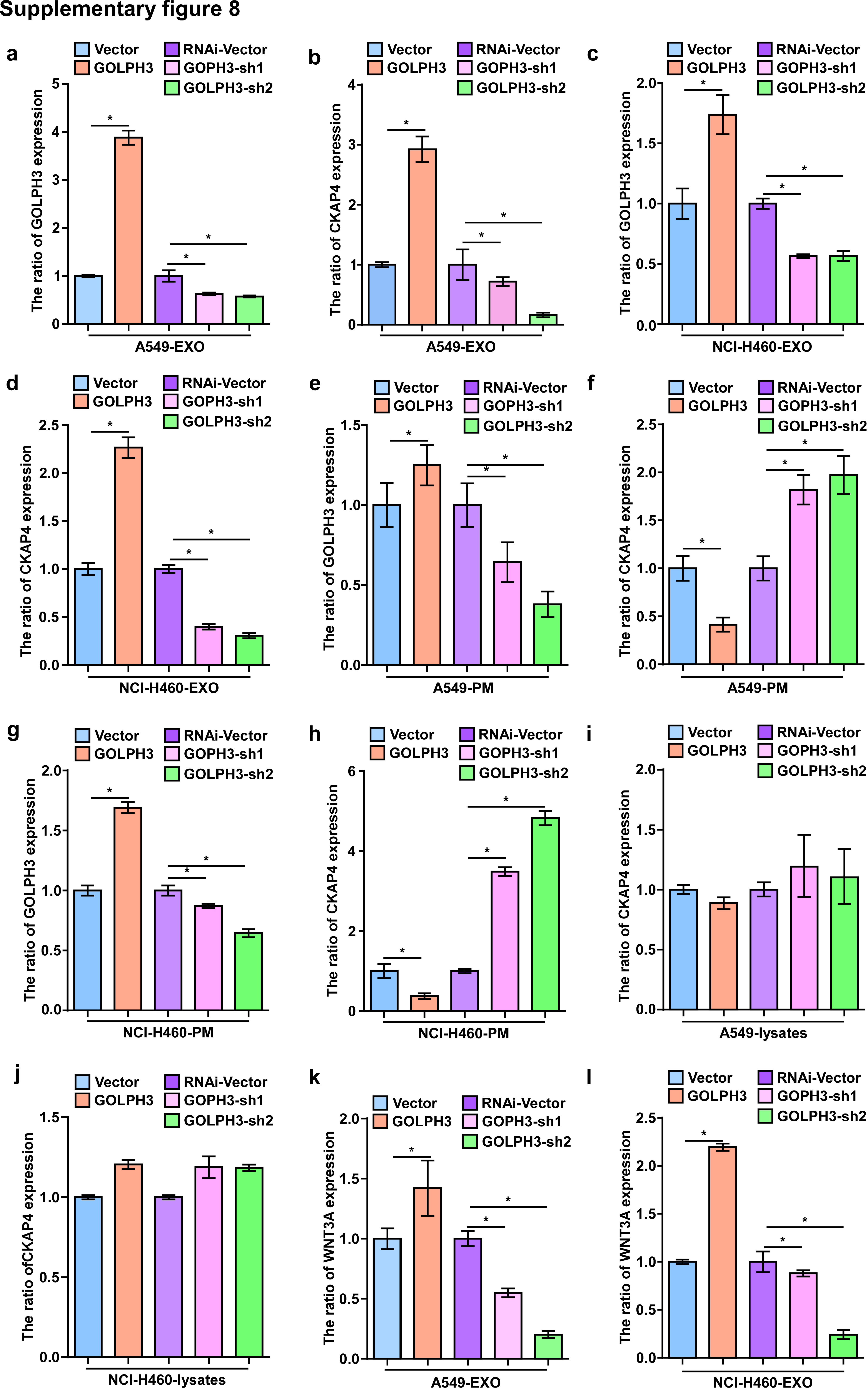

Supplement: Supplementary file 9 — Supplementary figure 8 [file 41419_2021_4265_MOESM9_ESM.tif]

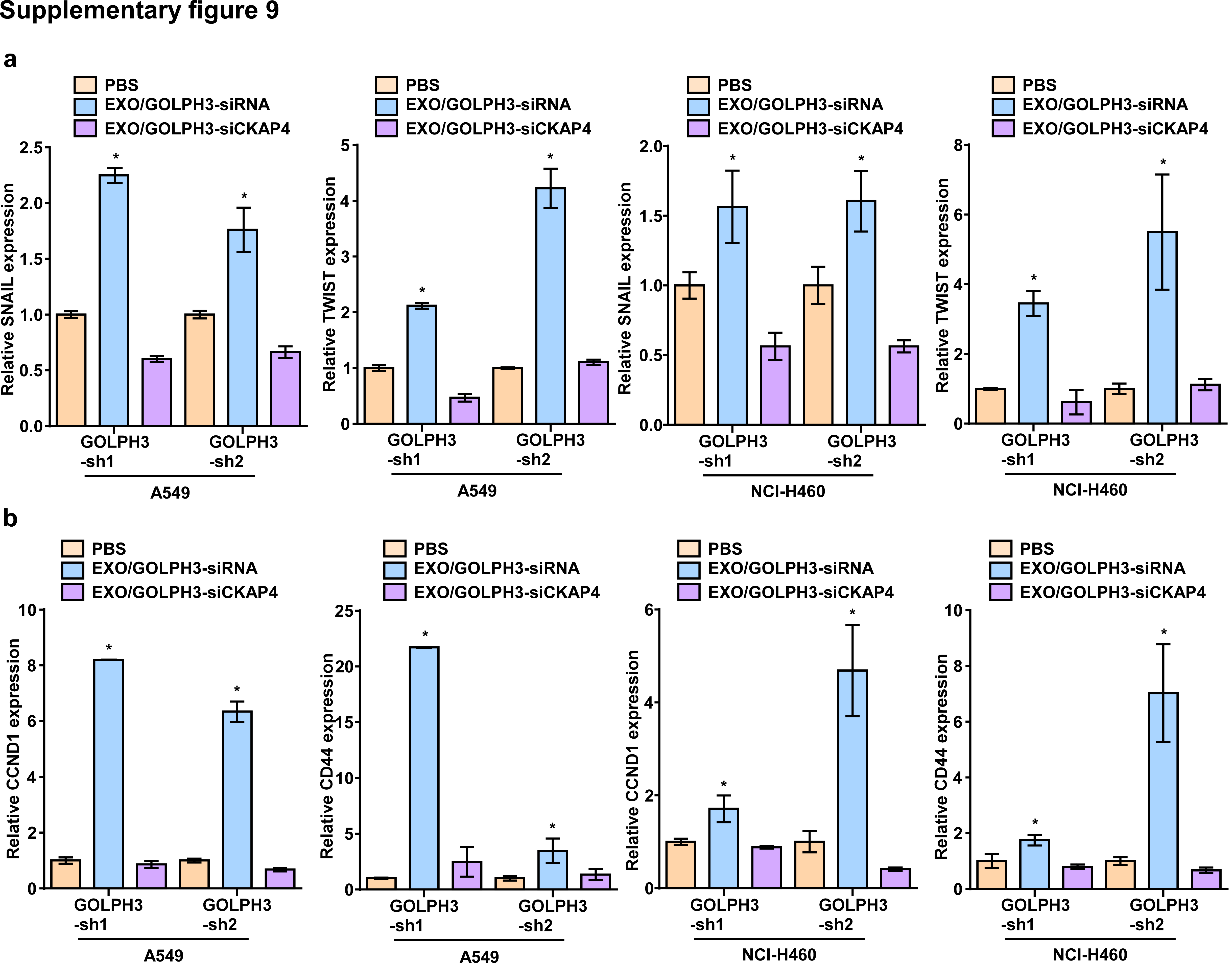

Supplement: Supplementary file 10 — Supplementary figure 9 [file 41419_2021_4265_MOESM10_ESM.tif]
