# Supplementary material for: GOLPH3/CKAP4 promotes metastasis and tumorigenicity by enhancing the secretion of exosomal WNT3A in non-small-cell lung cancer
Source: Cell Death Dis. 2021 Oct 21;12(11):976. doi: 10.1038/s41419-021-04265-8 (PMC8528870; doi:10.1038/s41419-021-04265-8)
Supplement: Supplementary file 11 — Supplementary table 1 [file 41419_2021_4265_MOESM11_ESM.docx]

**Supplementary table 1: Primers used for real-time PCR**

| Gene | Primers (5′-3′)-Forward: | Primers (5′-3′)-Reverse: |
| --- | --- | --- |
| GOLPH3 | ACATCCCCTCACCAATAACAAC | TAGCCAAATCATACTGCTCGTC |
| ABCG2 | AAGCCATTGGTGTTTCCTTG | CTGGATCCTGAGCCTTTGG |
| C-MYC | CGTCCTCGGATTCTCTGCTC | CTTCGCTTACCAGAGTCGCT |
| NANOG | ATGGAGGAGGGAAGAGGAGA | GATTTGTGGGCCTGAAGAAA |
| KLF4 | CCCCGTGTGTTTACGGTAGT | GAGTTCCCATCT CAAGGCAC |
| CCND1 | GCTGCGAAGTGGAAACCATC | CCTCCTTCTGCACACATTTGAA |
| CD44 | CGTGGAATACACCTGCAAAG | CGGACACCATGGACAAGTTT |
| TWIST | CGGGAGTCCGCAGTCTTA | CTTGAGGGTCTGAATCTTGCT |
| SNAIL | TCCAGAGTTTACCTTCCAGCA | CTTTCCCACTGTCCTCATCTG |
| GAPDH | ACATCCCCTCACCAATAACAAC | TAGCCAAATCATACTGCTCGTC |
