# Supplementary material for: GOLPH3/CKAP4 promotes metastasis and tumorigenicity by enhancing the secretion of exosomal WNT3A in non-small-cell lung cancer
Source: Cell Death Dis. 2021 Oct 21;12(11):976. doi: 10.1038/s41419-021-04265-8 (PMC8528870; doi:10.1038/s41419-021-04265-8)
Supplement: Supplementary file 12 — Supplementary table 2 [file 41419_2021_4265_MOESM12_ESM.docx]

**Supplementary table 2: Primers used for plasmid construction**

| Gene | Primers (5′-3′) |
| --- | --- |
| GOLPH3 | Forward: GACAAAGGCGCGCCTGAATTCATGACCTCGCTGACCCAGCGCAGCT |
|  | Reverse: GGGAGGGAGAGGGGCGGATCCTTACTTGGTGAACGCCGCCACCACC |
